# Fine‐scale environmentally associated spatial structure of lumpfish (*Cyclopterus lumpus*) across the Northwest Atlantic

**DOI:** 10.1111/eva.13590

**Published:** 2023-09-05

**Authors:** Barbara L. Langille, Tony Kess, Matthew Brachmann, Cameron M. Nugent, Amber Messmer, Steven J. Duffy, Melissa K. Holborn, Mallory Van Wyngaarden, Tim Martin Knutsen, Matthew Kent, Danny Boyce, Robert S. Gregory, Johanne Gauthier, Elizabeth A. Fairchild, Michael Pietrak, Stephen Eddy, Carlos Garcia de Leaniz, Sofia Consuegra, Ben Whittaker, Paul Bentzen, Ian R. Bradbury

**Affiliations:** ^1^ Northwest Atlantic Fisheries Centre, Fisheries and Oceans Canada St. John's Newfoundland and Labrador Canada; ^2^ AquaGen AS Trondheim Norway; ^3^ Department of Animal and Aquacultural Sciences, Faculty of Biosciences, Centre for Integrative Genetics Norwegian University of Life Sciences Ås Norway; ^4^ Department of Ocean Sciences, Ocean Sciences Centre Memorial University of Newfoundland St John's Newfoundland and Labrador Canada; ^5^ Maurice Lamontagne Institute, Fisheries and Oceans Canada Quebec Canada; ^6^ Department of Biological Sciences University of New Hampshire Durham New Hampshire USA; ^7^ USDA, Agricultural Research Service National Cold Water Marine Aquaculture Center Franklin Maine USA; ^8^ University of Maine Center for Cooperative Aquaculture Research Franklin Maine USA; ^9^ Centre for Sustainable Aquatic Research, Swansea University Swansea UK; ^10^ Marine Gene Probe Laboratory, Department of Biology Dalhousie University Halifax Nova Scotia Canada

**Keywords:** cleaner fish, environmental adaptation, genomics, population structure, spatial structure, whole‐genome re‐sequencing

## Abstract

Lumpfish, *Cyclopterus lumpus*, have historically been harvested throughout Atlantic Canada and are increasingly in demand as a solution to controlling sea lice in Atlantic salmon farms—a process which involves both the domestication and the transfer of lumpfish between geographic regions. At present, little is known regarding population structure and diversity of wild lumpfish in Atlantic Canada, limiting attempts to assess the potential impacts of escaped lumpfish individuals from salmon pens on currently at‐risk wild populations. Here, we characterize the spatial population structure and genomic‐environmental associations of wild populations of lumpfish throughout the Northwest Atlantic using both 70K SNP array data and whole‐genome re‐sequencing data (WGS). At broad spatial scales, our results reveal a large environmentally associated genetic break between the southern populations (Gulf of Maine and Bay of Fundy) and northern populations (Newfoundland and the Gulf of St. Lawrence), linked to variation in ocean temperature and ice cover. At finer spatial scales, evidence of population structure was also evident in a distinct coastal group in Newfoundland and significant isolation by distance across the northern region. Both evidence of consistent environmental associations and elevated genome‐wide variation in *F*_ST_ values among these three regional groups supports their biological relevance. This study represents the first extensive description of population structure of lumpfish in Atlantic Canada, revealing evidence of broad and fine geographic scale environmentally associated genomic diversity. Our results will facilitate the commercial use of lumpfish as a cleaner fish in Atlantic salmon aquaculture, the identification of lumpfish escapees, and the delineation of conservation units of this at‐risk species throughout Atlantic Canada.

## 1 INTRODUCTION

Cleaner fish, such as wrasses and the lumpfish (*Cyclopterus lumpus* Linnaeus, 1758) have been utilized as sea lice (*Lepeoptherius salmonis* and *Caligus elongates)* control measures in salmon aquaculture, in replacement of chemical therapeutants (Aaen et al., 2015; Lees et al., 2008; Riley et al., 2017). However, as the use of cleaner fish often involves the domestication and or translocation of wild individuals across regions to supply salmon farms (Jonassen et al., 2018; Treasurer, 2018), concerns exist regarding the implications of escapees into wild populations. Escape events of Atlantic salmon from aquaculture facilities have been repeatedly documented (Glover et al., 2012; Wringe et al., 2018) with subsequent studies demonstrating hybridization and introgression (Bolstad et al., 2021; Bradbury et al., 2022; Glover et al., 2020; Wringe et al., 2018), life history changes (Besnier et al., 2022; Bolstad et al., 2017), or population decline in the wild (Sylvester et al., 2019). For cleaner fish, recent research documenting escapees raise the possibility of similar impacts of escaped cleaner fish on wild populations, although little research has been done in this area to date. A study found gene flow between aquaculture and wild populations of *Symphodus melops* (Corkwing Wrasse), indicating that escapees were hybridizing with wild populations, which could lead to the erosion of population structure and alteration of the genetic composition of wild populations (Faust et al., 2018). A similar result was also observed in another cleaner fish species, *Ctenolabrus rupestris* (Goldsinny Wrasse; Jansson et al., 2017), indicating escape and hybridization may occur frequently. As such, a robust understanding of genetic diversity of wild cleaner fish is essential to the development and usage of these species in salmon aquaculture, and to better manage the impacts of escapees via introgression into wild populations.

Lumpfish are a semi‐pelagic teleost widely distributed throughout the North Atlantic Ocean (Figure 1 from Davenport, 1985; Powell et al., 2018). Adult lumpfish swim inshore to spawn and generally return to the same spawning area repeatedly (within 80 km) (Kennedy et al., 2015; Kennedy & Olafsson, 2019), suggesting some degree of homing behavior. Once hatched, larvae attach to nearby substrates (Ingolfsson, 2000) and remain in shallow inshore areas for up to a year before migrating out to sea for the duration of their adult life (aside from returning inshore to spawn). Lumpfish largely prefer cold water, high‐salinity habitats but also show adaptation to tolerating more extreme conditions, such as warmer water in the English Channel, and lower salinity in Hudson Bay and the Baltic Sea (Davenport, 1985; Hedeholm et al., 2017; Powell et al., 2018). Overall, lumpfish have broad habitat preferences and occupy a wide niche (Garcia de Leaniz et al., 2022). In Atlantic Canada, lumpfish have declined by ~58% over the last two decades in the core portion of their range around southern Newfoundland possibly due to changes in sea water composition (temperature, salinity, etc.), degradation of suitable habitat (e.g., eelgrass for juvenile stage or nesting sites), and/or overfishing of spawning females in a roe fishery (COSEWIC, 2017; Simpson et al., 2016) raising clear conservation and management concerns for this species.

**FIGURE 1:**
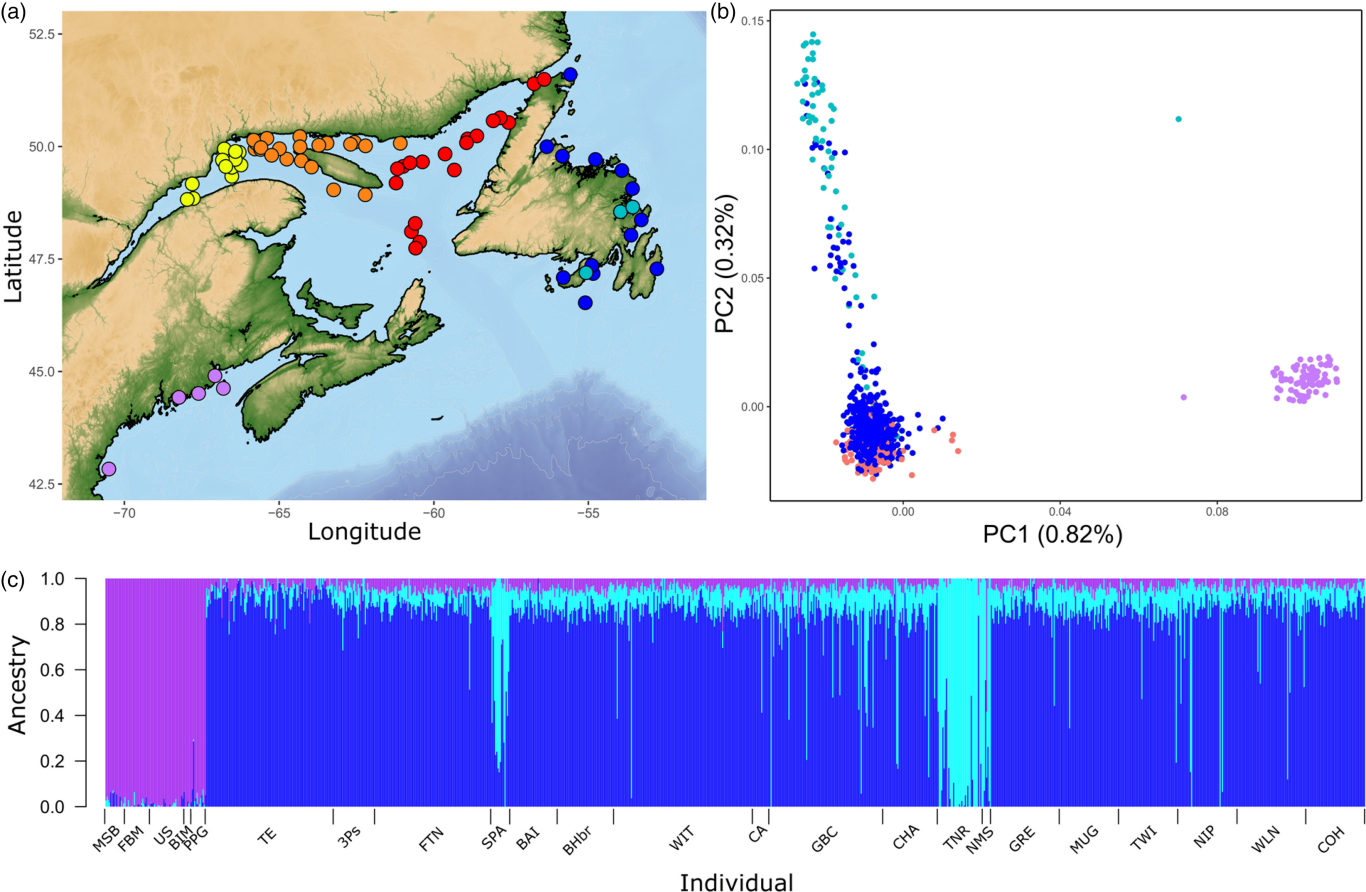
(a) Distribution map of sampling locations, (b) Principal components analysis (PCA) of neutral loci from the SNP array, and (c) Admixture plot where *K* = 3, of all lumpfish individuals across North America using the 70K SNP array data. The colors represent different locations and are consistent from plot to plot, where dark blue is Newfoundland adults, cyan is Newfoundland juveniles, purple is the southern sites (Grand Manan and Gulf of Maine), and the Gulf of St. Lawrence (GSL) is separated into western (yellow), central (orange), and eastern (red) regions in the map. Additionally, the GSL region as a whole is represented in the PCA by orange points and was not a distinct group from Newfoundland adults in the admixture analysis (labeled TE). See Supporting information S1 for site names associated with abbreviations in admixture plot.

In marine systems, high gene flow is often expected due to the lack of physical barriers and high egg and larval dispersal potential (Bradbury et al., 2008; Palumbi, 1994). However, examples of marine species which do not fit this paradigm and display evidence of regional scale neutral or adaptive genetic structure are accumulating (Bernatchez et al., 2017; Funk et al., 2012; Kess et al., 2020; Kess, Dempson, et al., 2021, Kess, Einfeldt, et al., 2021; Luikart et al., 2003; Mares‐Mayagoitia et al., 2021; Miller et al., 2019). Genomic studies across a range of marine taxa suggest regional diversity associated with variation in marine climate such as temperature extremes (e.g., Stanley et al., 2018) consistent with the presence of adaptive divergence within marine species (Hauser & Carvalho, 2008). Primarily using microsatellite loci, previous studies have identified the West Atlantic lumpfish (USA and Canada) as a single distinct group from lumpfish found elsewhere (Pampoulie et al., 2014; Whittaker et al., 2018), supporting a single designatable unit (DU; discrete and evolutionarily significant unit) across their Canadian range (COSEWIC, 2017). However, a separate study investigating different sites in Greenland, also using microsatellite markers, uncovered two distinct groups (a north and a south group in Greenland; Garcia‐Mayoral et al., 2016) indicating that fine‐scale genetic structuring could occur in lumpfish elsewhere as well, however, the source of this divergence remains unclear. Given the growing commercial interest and conservation concern surrounding this species, a high‐resolution genomic analysis of spatial structure is currently warranted to quantify the scale of differentiation and diversity present to inform the development of cleaner fish applications using lumpfish.

Previous studies have provided poor genomic and geographic resolution of population structure of this commercially important cleaner fish throughout their North American range. Therefore, our goal was to robustly assess the extent of spatial population structure and environmental adaptation of lumpfish throughout the Northwest Atlantic. Specifically, our objectives were to: (1) evaluate broad and fine‐scale population structure of lumpfish in the Northwest Atlantic using whole‐genome re‐sequencing (WGS) and SNP array data, with the goal of identifying if lumpfish are actually one population, which is the current assessment (Simpson et al., 2016), or uncover if multiple populations (i.e., DUs) are present; (2) use these data to quantify environmental associations with genetic structure and determine the genomic distribution of environmentally associated variation, thereby evaluating the potential for environmentally associated adaptation in this species across Atlantic Canada; and finally (3) provide a comparison of the WGS and SNP array datasets and their resolution of spatial structure. As the interest and utilization of lumpfish as a cleaner fish in salmon aquaculture intensifies, this work will inform the continued management and conservation of this at‐risk species throughout the region and more broadly (Lorance et al., 2015).

## 2 METHODS

### 2.1 Sample collection and genotyping

We sampled 1115 individuals from 79 locations throughout the North Atlantic from 2009 to 2019 (Figure 1a; Supporting information S1) in conjunction with scientific research surveys (trawl and beach seines) and commercial fishing activities. Sampling locations included waters around Newfoundland (*N* = 994), the Gulf of St. Lawrence (GSL) (*N* = 108), waters around Grand Manan (*N* = 13), and from the Gulf of Maine (*N* = 83). Due to the different sampling approaches utilized, differences in the sexes and life stages collected were present. Research vessel surveys collected individuals using a bottom trawl and generally targeted adults of both sexes, commercial samples were largely adult females due to the selective nature of the fishery for roe, and coastal sites around Newfoundland (TNR, SPA, and NMS, Figure 1a), sampled using a beach seine were juvenile lumpfish comprising both sexes. In all cases, fin clips were preserved in 95% ethanol for subsequent DNA extraction.

For the 70K SNP array data, DNA extractions were performed using DNeasy Blood and Tissue kits (Qiagen) according to manufacturer's protocols. Genomic DNA was visualized by 1% agarose gel electrophoresis and quantified using Quant‐iT PicoGreen ds‐DNA Assay kits (Thermofisher) on a fluorescent plate reader. Genomic DNA was normalized to 15 ng/μL and sent to the Centre of Integrative Genomics (CIGENE) for genotyping on the lumpfish 70K SNP Affymetrix Axiom array (produced by CIGENE and Aquagen; Holborn et al., 2022).

We conducted medium coverage (~8×), whole‐genome re‐sequencing (WGS) of 848 lumpfish from multiple sites across North America (Figure 1a; Supporting information S1). The same individuals were used for WGS as in the array; however, individuals from sites US1, US2, CA, and BHbr, were only present with array data. For whole‐genome re‐sequencing library preparation, DNA extraction followed Mayjonade et al. (2016), using Longmire's buffer (Longmire et al., 1997) for tissue lysis. Library preparation followed a modification of the protocol from Therkildsen and Palumbi (2017) by scaling down Nextera DNA Flex Library Prep Kits (Illumina) reaction volumes to 0.13× then used a standard volume in the Illumina protocol. We then used Kapa Hi‐Fidelity Library Amplification Kits (Roche) for library amplification (20 μL reactions, 4 μL Nextera Unique Dual Indexes Set A (Illumina)). Libraries were quantified by Qubit (ThermoFisher) and an Agilent Bioanalyzer was used to check average fragment size. Libraries were normalized from 96‐well plates to equimolar concentrations and pooled separately for each lane of sequencing. We sequenced pooled libraries at The Genome Quebec Centre d'expertise et de services on 11 lanes of an Illumina NovaSeq6000 S4.

### 2.2 SNP array filtering

The SNP array data was mapped to the North American lumpfish genome (Holborn et al., 2022). SNP data was transformed from .ped/.map files into .bed/.bim/and .fam files using Plink v1.9 (Chang et al., 2015), and sample/SNP level filtering was performed as quality control; we filtered the SNP set by individuals with high genotyping (*mind* 0.05), sample call rates (*geno* 0.05), and minor allele frequency (*maf* 0.01). SNPs that did not align to chromosomes were filtered out using the *not‐chr* function in Plink. We also filtered individuals by relatedness in Plink, using the *genome* function and a pi‐hat threshold of 0.2 (second‐degree relative) in order to remove any false signals of population structure as the highly related individuals could cluster as distinct groups.

### 2.3 Bioinformatic processing of whole‐genome data

Whole‐genome library quality was assessed using FastQC, (Andrews, 2010). We used fastp 0.23.1 (Chen et al., 2018), parallelized with GNU parallel (Tange, 2011), with default parameters to remove low‐quality bases and adapter content. We aligned trimmed reads from each individual to the Common lumpfish primary curated reference genome (Holborn et al., 2022) reference assembly (GenBank Accession: GCA_009769545.1) using bwamem2 (Li, 2013). We then followed best practice recommendations for Genomic Analysis Toolkit (DePristo et al., 2011): we used the GATK 4.2. *MarkDuplicates* function to remove duplicated reads, which were then realigned around potential insertions and deletions using *RealignerTargetCreator* and *IndelRealigner* functions in GATK 3.7.

To account for uncertainty inherent in base‐calling from genome re‐sequencing, we directly estimated genotype likelihoods using Analysis of Next Generation Sequencing Data (ANGSD 0.935; Korneliussen et al., 2014). For each chromosome, we exported genotype likelihoods in beagle format (Browning & Browning, 2007) for all SNPs passing quality filers (‐*minMapQ* 30 ‐*minQ* 20 *‐SNP_pval* 2e‐6 *‐uniqueOnly* 1 *‐remove_bads* 1), with >80% (‐*minInd* 870) of individuals genotyped and a minimum of 1000 reads were present per locus (*‐setMinDepth* 1000). We also exported called genotypes for each SNP as .bcf files using the ‐*dobcf* flag, and converted to .vcf files (Danecek et al., 2011) using bcftools 1.11 (Li et al., 2010). We assessed mean depth by estimating sequence coverage across the largest chromosome using vcftools 0.1.16. We imputed and phased .vcf files using genotype likelihood information with Beagle 4.0 (Browning & Browning, 2007, 2016), resulting in 5,008,312 SNPs with a mean coverage of 8× for downstream analysis.

### 2.4 Basic statistics

We had a total of 58 different sampling sites across the entire GSL region (Figure 1a; Supporting information S1) with only one and nine individuals per site. Therefore, to use the GSL samples in our dataset, grouping of individuals was required as many analyses can be biased by small samples sizes (Lin, 2018). Using a combination of the Northwest Atlantic Fisheries Organization (NAFO) region and individual‐level analyses (neighbor joining tree, PCA, and admixture), we determined the best geographic groupings of individuals for subsequent analyses (see Supporting information S2 for detailed methods on how we determined groups). However, as there was little to no structure across this region, we mainly relied on grouping based on NAFO region. Regardless of the method for pooling samples, there could be a potential bias on the resolution of spatial structure, as any level of pooling could erode variation that exists in each (or specific) sample sites. However, for the purpose of this study, and given the lack of structure in the region, this was deemed tolerable. Using both datasets (SNPs within exons only for WGS data), estimates of observed and expected heterozygosity (*H*_O_ and *H*_E_, respectively) were performed using Arlequin v3.5 (Excoffier & Lischer, 2010) and we identified the inbreeding coefficient (*F*_IS_) within each site using the *ibc* function in Plink.

### 2.5 Population structure analysis

To further explore genetic variation between all individuals within the 70 K SNP array and WGS data, we generated a principal component analysis (PCA) using the pcadapt package (Luu et al., 2017) to calculate PC scores for *K* = 10 and min.maf of 0.01, and the *plot* function in R (R Development Core Team, 2016) to visualize the data. Here, we used neutral and outlier loci separately to ensure signatures of selection were not biasing the analysis. We found outlier loci by first running a PCA using the optimal number of *K* (determined by visualizing values of *K* in a screeplot) and extracted *p‐*values. We then corrected *p‐*values using the *qvalue* package (Storey et al., 2020) to control for false discovery rate (Storey & Tibshirani, 2003). Loci with *q‐*values under 0.05 were considered outlier loci. We visualized *q‐*value loadings as a Manhattan plot, which we constructed using the *qqman* package (Turner, 2014).

We used Admixture v1.3.0 (Alexander et al., 2009) to estimate ancestry coefficients and assignment of individuals into groups for SNP array data. We determined the best level of structural clusters (*K*) present by choosing the lowest cross validation value. This analysis is sensitive to linked loci, therefore, prior to determining the optimal *K* value we filtered the datasets by LD in Plink using the function *indep‐pairwise*, with a window size of 50, a step size of 5, and a pairwise *r* ^2^ threshold of 0.5. For WGS data, we used the admixture proportion estimation function in PCANGSD, and plotted individual Q values for the highest supported *K* inferred by PCANGSD using the MAP algorithm.

We used the 70K SNP array data to calculate a neighbor joining tree using a Nei's D distance matrix in the R package *StAMPP* (*stamppNeisD*). The *ape* package (*nj*) was used to build the tree using default settings and FigTree (Rambaut, 2010) was used to visualize the phylogeny. Using both datasets, we calculated pairwise *F*_ST_ in Arlequin and visualized the data using the *ggplot* package in R. Populations with small sample sizes could potentially lead to an overestimation of genetic differentiation between populations, therefore, locations with <10 individuals were removed from this analysis. We calculated isolation by distance (IBD) by comparing geographic distance between sites to pairwise *F*_ST_ scores generated in the step above. The geographic distance between all sampling sites were determined with the least path method implemented in package *marmap* v1.0.5 (Pante & Simon‐Bouhet, 2013). The package *ggplot* was used to visualize the data in R.

### 2.6 *F*_ST_ across the genome and gene enrichment

To identify which regions of the genome were responsible for differentiation between different structural clusters, using the 70K SNP array data we focused on individuals with >0.75 admixture membership to a cluster from *K* = 2 plots. Depending on the size of different groups, we randomly down sampled to keep both groups equal. We used the *fst* function in Plink to estimate the *F*_ST_ values of each SNP across the genome between each cluster. We constructed a Manhattan plot in R using the package *ggman* (https://github.com/drveera/ggman). To identify patterns of genomic divergence between clusters identified by PCA for the WGS data, we carried out pairwise *F*_ST_ scans of 50 individuals selected from each cluster using the *weir‐fst‐pop* function in *vcftools*, and filtering for a minor allele frequency of 0.05 in the reduced set of individuals. We estimated Weir and Cockerham's weighted *F*_ST_ (Weir & Cockerham, 1984), as well as per‐locus *F*_ST_, for each comparison.

### 2.7 Signatures of environmental adaptation

Finally, we explored associations between environmental and genomic variation. We selected a set of uncorrelated environmental variables (*r* ^2^ < 0.7 using all marine databases in *sdmpredictors* package in R; see Supporting information S3 for correlated variables) comprising climate and oceanographic features important to different habitats across the Northwest Atlantic (e.g., such as temperature and ice cover variables). We used a Pearson correlation to further reduce to eight uncorrelated bioclimatic variables using the *psych* package (*pairs. panels*; see results section for variables). We followed up by running a variance inflation factor (vif) scores model to verify which climatic variables had a high degree of multicollinearity, where a value <10 indicated low multicollinearity (Zuur et al., 2010). We tested for a relationship between genomic and environmental variation using redundancy analysis (RDA) using the *rda* function in *vegan* (Oksanen et al., 2020). We carried out an RDA for all samples across North America to test for range wide environment driven population structure. We carried out another RDA restricted to Newfoundland samples to test for fine‐scale environmentally associated structuring. We then used the *anova.cca* function with 999 permutations to assess significance of environmental variables for each model. Additionally, we used a conditioning (*Z*) matrix composed of Moran's Eigenvectors Maps (MEM) to correct both RDAs for geographic space using the package *adespatial* (*mem* function) in R (Dray et al., 2023). The first two MEMs were used as a conditioning matrix within the RDA (*rda* function in *vegan*). To reduce computation time with the WGS data, we down sampled genotypes to only include SNPs from genomic regions overlapping exons (i.e., exome) using *bedtools intersect*. To identify genomic regions significantly associated with environmental variation, we used the Mahalanobis distance outlier detection approach implemented in the *RDadapt* R function described in Capblancq et al. (2018). Using the Entrez server we recorded the genes the SNPs were within as well as the general gene ontology (GO) information, identified by the Metascape web server (Zhou et al., 2019).

### 2.8 Direct comparison between the array and WGS

Admittedly, the two independent datasets have differing tradeoffs or potential biases; the SNP array is subject to potential ascertainment bias as it was constructed using Norwegian lumpfish (Knutsen et al., 2018), while the coverage of WGS data exhibits individual level and site level quality and coverage variation. To compare, we used the same individuals from each dataset and calculated per locus *F*_ST_ using the northern and southern groups inferred from PCA as our two comparisons in Plink using the *fst* function, where the values were averaged across loci. For the WGS data, we used a windowed approach using *windowscanR* in R to generate per locus measures of *F*_ST_: using 50, 100, and 200 kilobases (kb) as our windows and a 1 kb step size between windows using a reduced set of whole‐exome data for computational efficiency (244,669 SNPs). We filtered out any windows with less than three observations and calculated the outlier cutoff for each run of the different window sizes (i.e., three standard deviations away from the *F*_ST_ mean for a given window run). We generated pairwise *F*_ST_ values between sites in Arlequin for both datasets. We then calculated discordance between estimates of *F*_ST_ using a Pearson correlation (*cor.test* function in R). We also ran correlations between the first two PC axes, as well as between the three admixture groups that were generated previously for the entire North American dataset, also using the *cor.test* and a linear regression (*lm* function in R).

## 3 RESULTS

### 3.1 Genetic diversity

Across North American sites, there were a total of 1064 individuals with a set of 63,819 filtered SNPs from the 70 K SNP array data and 848 individuals with a set of 5,008,312 filtered SNPs from the WGS data. We had 20 sites from Newfoundland (*N* = 873 from the SNP array/678 from WGS), 53 sites from the GSL (*N* = 108/106), one site from the Bay of Fundy (*N* = 13/11), and five sites from the Gulf of Maine (*N* = 70/53). Based on NAFO regions and individual‐based analyses (see Supporting information S2 for details), we were able to cluster the GSL samples into three regional groups: western GSL (TE1), central GSL (TE2), and eastern GSL (TE3) (Figure 1a; Supporting information S2 for map of the GSL sites placed into the same groups). These new groups were used in all analyses moving forward. Based on the 70K SNP array data, we found the observed/expected heterozygosity (*H*_O_/*H*_E_) overall was 0.293/0.292 and ranged from 0.259/0.259 in FTN, Newfoundland, Canada to 0.357/0.356 in BIM, Gulf of Maine, USA (Table 1, Supporting information S1). *F*_IS_ values across sites were close to zero and there were no significant deviations. However, seven sample sites had small positive values: BHbr, CA, CHA, SPA, TNR, WLN, and PPG (Table 1).

**TABLE 1:** Summary statistics for sampled lumpfish populations based on the 70K SNP array data.

Area	Population code	Sample size (*N*)	Nucleotide diversity	Observed heterozygosity (*H*_O_)	Expected heterozygosity (*H*_E_)	Inbreeding coefficient (*F*_IS_)
Newfoundland	BAI	55	0.250032	0.27527	0.27539	−0.00434
	BHbr	48	0.251557	0.27839	0.27906	0.00163
	CA	14	0.252546	0.31838	0.31957	0.00265
	CHA	46	0.250769	0.27838	0.27908	0.00139
	COH	50	0.247906	0.27472	0.27476	−0.00276
	FTN	98	0.243408	0.25906	0.25916	−0.00156
	GBC	98	0.245966	0.26102	0.26172	−0.00085
	GRE	58	0.247237	0.27122	0.27121	−0.00319
	MUG	50	0.249477	0.27563	0.27562	−0.00198
	NIP	50	0.246448	0.27357	0.27383	−0.00052
	NMS	10	0.248073	0.33628	0.32732	−0.03008
	S3P	35	0.250850	0.28544	0.28580	−0.00007
	SPA	22	0.243253	0.30715	0.30856	0.00346
	TNR	50	0.240823	0.28039	0.28084	0.00083
	TWI	50	0.251762	0.27731	0.27748	−0.00072
	WIT	118	0.249807	0.26329	0.26325	−0.00217
	WLN	59	0.249635	0.27262	0.27325	0.00150
New Brunswick	PPG	13	0.261539	0.32520	0.32879	0.01105
United States	BIM	8	0.260507	0.35650	0.35566	−0.00299
	FBM	29	0.260339	0.30436	0.30409	−0.00154
	MSB	17	0.259171	0.31654	0.31621	−0.00554
	US1	15	0.262572	0.32480	0.32262	−0.00747
	US2	14	0.258436	0.32580	0.32428	−0.00754

*Note*: The Gulf of St. Lawrence populations were excluded due to their low sample sizes.

### 3.2 Population structure

Outlier analysis revealed a total of 3919 *q‐*value corrected outlier loci in the SNP array dataset and a total of 123,383 loci in the WGS dataset (Supporting information S4 for outlier loci list). PCAs of both neutral and outlier loci revealed large‐scale division, with the first axis clearly dividing individuals from the southern sites (the Bay of Fundy and the Gulf of Maine) from the northern sites (Newfoundland and the GSL; Figure 1b). The second axis additionally separated the north into two groups: a primary group, encompassing all the GSL and most Newfoundland individuals, and a secondary group, encompassing most juveniles and some adult individuals from a variety of sites across Newfoundland. These three groupings of individuals were additionally verified by *k*‐means clustering (Supporting information S5). The primary and secondary axes were able to explain 4.67/0.82% (outlier/neutral) and 1.75/0.32% (outlier/neutral), respectively, of the variation in the SNP dataset, while the PCA of the WGS was 0.60/0.44% (outlier/neutral) and 0.41/0.39% (outlier/neutral), for axis one and two, respectively. The PC loadings identified large peaks on chromosomes 11, 16, 17, and 22 for the outlier loci, and a large peak on chromosome 5 and a small peak on chromosome 9 for the neutral loci (Supporting information S6).

A PCA plot of all southern sites alone revealed no evidence of discrete groups on any of the first six axes (for the neutral or outlier loci, see Supporting information S4 for list of outlier loci), however, there was a small geographic organization of sites along axis one for neutral loci and axis two for outlier loci (Supporting information S7a,b). The site, US1, appeared to be dissimilar from the rest, while other individuals were loosely organized by site, however, the organization did not appear to be related to geographic proximity. PCAs of sample sites from the primary northern group (based on *k*‐means clustering from Supporting information S5) did not reveal any obvious discrete clusters with the neutral or 1029 outlier loci (Supporting information S4 and S7c,d); however, there was some organization of sites along the primary axis using neutral loci and along the tertiary axis using outlier loci. Individuals from sites in the GSL range were partially separated out from Newfoundland individuals, with all Newfoundland individuals clustering loosely by geographic distance.

Admixture results were similar to those from the PCAs. Using cross validation values of *K* = 1 to 20, the best *K* was determined to be 3; this result was identified for the array data as well as the WGS data. The south/north division was evident as all individuals from the south had a high proportion of membership to the same group, with individuals outside of that range having very low assignment into that group (Figure 1c). Within the northern cluster there was further evidence of subdivision as in the PCA with one group comprised of individuals from the GSL and Newfoundland, and a second group limited largely to three Newfoundland locations (i.e., TNR, SPA, and NMS). To identify fine‐scale structure that may have been masked by high genetic differentiation between northern and southern sites, we re‐ran Admixture on each of the three groups individually. Admixture analysis of (1) southern sites and (2) secondary northern sites did not reveal any additional structuring (*K* = 1); however, an Admixture plot of the (3) primary north group revealed two main clusters, with all Newfoundland individuals belonging to one, and some members of the GSL (specifically from the west and central GSL range), belonging to a second (Supporting information S7e).

A neighbor joining tree discretely divided the north and south sample sites (Supporting information S8). Within the southern group, the Gulf of Maine branched monophyletically, with the one site from the Bay of Fundy sitting at the end of the monophyletic cascade. Within the north, the juvenile sites comprised a discrete cluster and had similar branch lengths compared to the southern sites, possibly indicating large genetic distances between these juvenile sites and other sites in the northern range.

Population pairwise *F*_ST_ values ranged from <0.001 to 0.045 between all samples (Supporting information S9a). Most sites were significantly differentiated from each other with the largest *F*_ST_ values between northern and southern sites (*F*_ST_ = 0.029 and *p*‐value <0.001 with a 95% confidence interval (CI) +/−<0.001 (Supporting information S10) for all northern sites compared to southern sites). However, pairwise *F*_ST_ comparisons across the northern sites (both the primary and secondary group) appeared to not be associated with geographic distance. Despite inconsistent isolation by distance within the north range, we observed significant isolation by distance across the entire study area (*F* statistic = 424.2, *p*‐value <0.0001, multiple *R* ^2^ = 0.608; adjusted *R* ^2^ = 0.606; Supporting information S9b).

When we re‐ran pairwise *F*_ST_ across the primary northern group, we found sites that were further apart generally had higher *F*_ST_ values (Supporting information S9c), implying the secondary group in Newfoundland was responsible for the incongruence seen in the previous *F*_ST_ analysis. With the secondary group removed, we also found a strong, positive isolation by distance relationship between sites within the primary northern group (*F* statistic = 354.4, *p*‐value <0.0001, multiple *r* ^*2*^ = 0.701; adjusted *r* ^*2*^ = 0.699), in which 69.9% of the data were explained by the distance between sites (Supporting information S9d). The sites in Newfoundland were generally more differentiated from all the GSL sites, although more so between the east and central GSL range. With the GSL removed, we observed a slightly weaker IBD; however, Newfoundland sites (with secondary group individuals removed) was significantly structured by distance (*F* statistic = 31.6, *p*‐value <0.0001, multiple *r* ^*2*^ = 0.235; adjusted *r* ^*2*^ = 0.227; Supporting information S9e). An IBD analysis of only southern sites was not significant (*F* statistic = 0.010, *p*‐value = 0.922, multiple *r* ^*2*^ = 0.0007; adjusted *r* ^*2*^ = −0.076).

### 3.3 *F*_ST_ across the genome and gene enrichment

**FIGURE 2:**
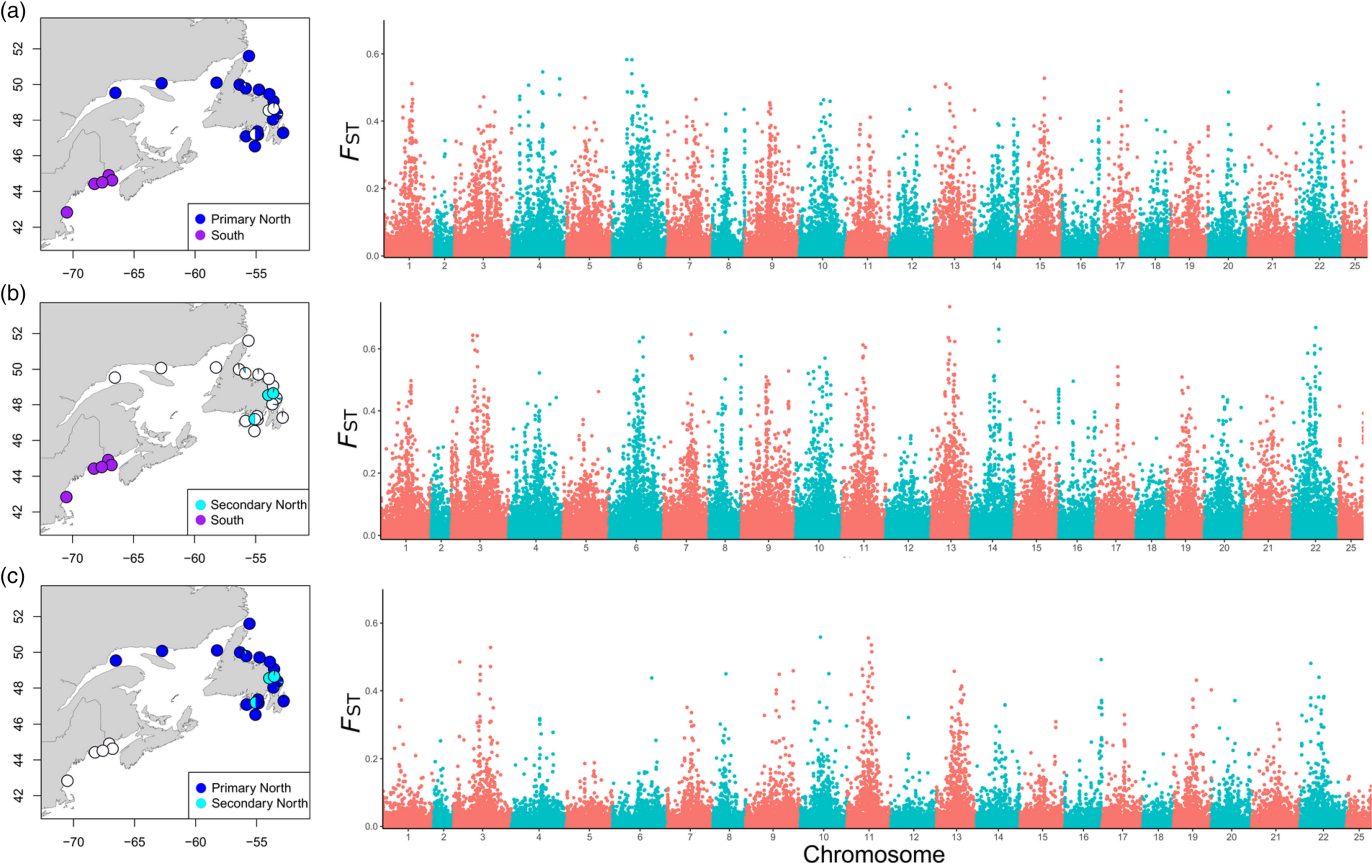
*F*_ST_ across the genome of the 70 K SNP array data where (a) is the south region compared to the primary north group, (b) is the south region compared to the secondary north group, and (c) is the comparison between the primary and secondary north groups. Maps on the left indicate the location of the groups being compared. Open white circles represent sampling locations that were not used in the comparison.

Using the SNP array dataset, we found 882 individuals belonging to the primary northern group and 52 individuals in the secondary northern group, based on *K* = 2 (over 0.75 membership). We compared the areas of differentiation across the genome between the two northern groups and the south group and found highly significant, genome‐wide *F*_ST_ differentiation between each comparison (Figure 2). Every chromosome had SNPs with high *F*_ST_ values, with the top SNPs over 0.5. The mean *F*_ST_ across the genome between the southern: and (1) primary northern group was 0.026 (*p*‐value <0.001), and (2) secondary northern group was 0.034 (*p*‐value <0.001). The mean *F*_ST_ between the primary northern and secondary group was 0.012 (*p*‐value <0.001). In particular, chromosomes 3, 9, 10, 11, 13, and 22 contained the greatest proportion of outliers (Figure 2c).

We uncovered 315 outlier 10 Kbp windows with elevated PC1 scores and *F*_ST_ between northern and southern groups both exceeding the 99th percentile, overlapping 149 genes with Entrez gene symbols. However, there was no evidence of significant enrichment among these genes using entrez IDs in the metascape web server. Comparing the primary and secondary north groups, we identified 313 outlier windows with elevated PC1 and *F*_ST_ scores. These windows overlapped with 182 genes with Entrez gene symbols, but again did not exhibit pathway or process‐specific enrichments.

### 3.4 Signatures of environmental adaptation

**FIGURE 3:**
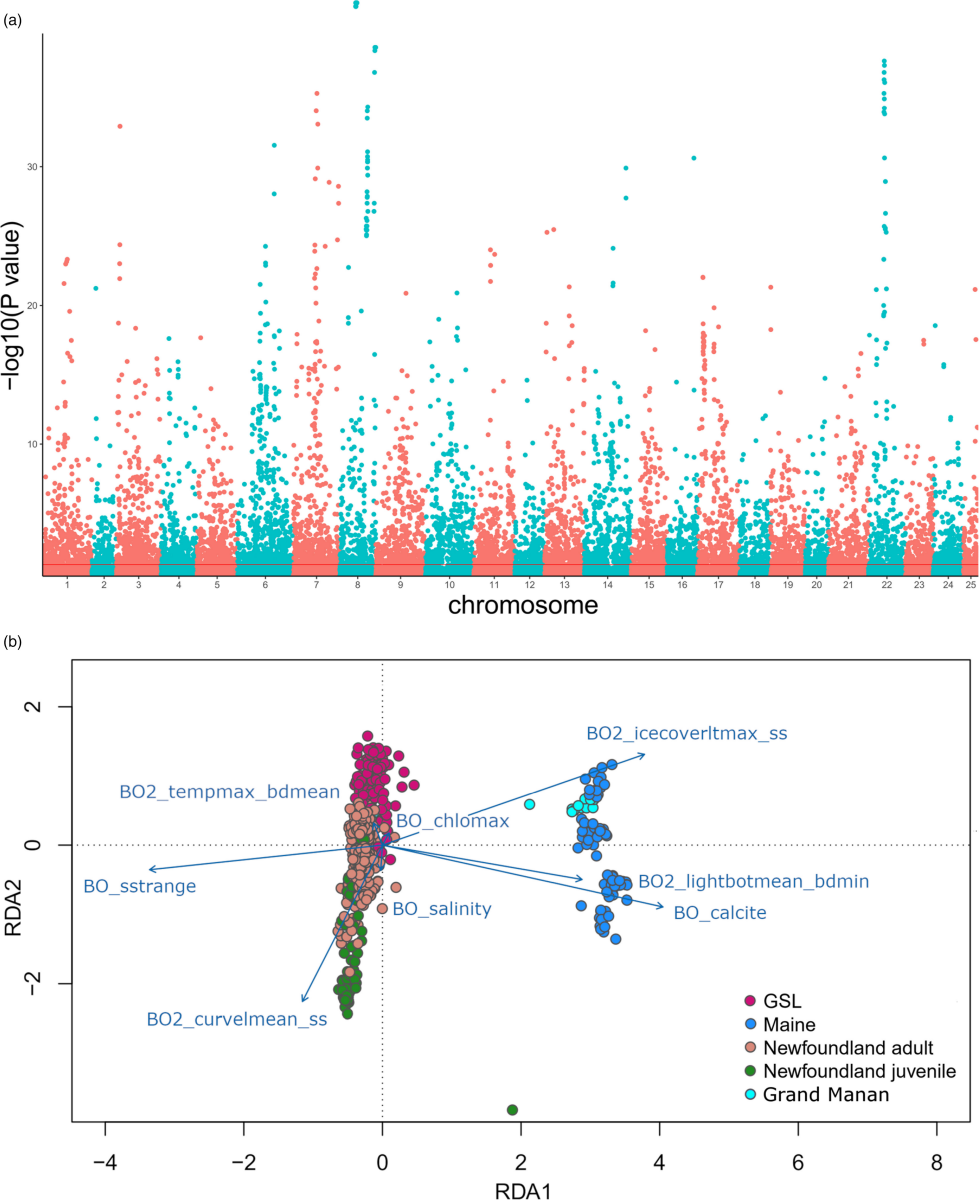
A redundancy analysis (RDA) of all North America individuals using the whole‐genome re‐sequencing (WGS) data where (a) is a Manhattan plot of the loadings where the red line indicates the threshold in which values above are the top 1% of loci, and (b) is the ordination plot of the first two RDA axis. The *r* ^2^ of the model was 0.011, suggesting the constrained ordination explained approximately 1.1% of the genetic variation. The points represent the individuals colored by their population of origin, while the blue arrows represent the environmental predictors. The relative arrangement of the points and arrows on the plot represents their relationship with the RDA ordination axes. In this plot, the Gulf of St. Lawrence is abbreviated to GSL.

Based on an analysis of correlation among environmental variables for all sites in this study, eight independent variables were associated with outlier loci: BO_calcite (669 SNP array loci/2198 WGS loci), BO_chlomax (96/1380 loci), BO_salinity (87/1025 loci), BO_sstrange (184/391 loci), BO2_tempmax_bdmean (38/446 loci), BO2_lightbotmean_bdmin (290/1923 loci), BO2_icecoverltmax_ss (331/920 loci), and BO2_curvelmean_ss (284/1145 loci; Figure 3a). It is important to note, these variables represent the environmental factors regarded as most important in this system, and however, each variable has a group of highly correlated additional variables (see Supporting information S3 for correlated variables). Additionally, we corrected for geography using MEM1 and MEM2 as conditions in the RDA, and identified outlier loci on the same variables: BO_calcite (367 SNP array loci/2030 WGS loci), BO_chlomax (33/1840 loci), BO_salinity (56/1225 loci), BO_sstrange (283/227 loci), BO2_tempmax_bdmean (177/513 loci), BO2_lightbotmean_bdmin (317/1888 loci), BO2_icecoverltmax_ss (341/688 loci), and BO2_curvelmean_ss (221/1230 loci; Supporting information S11). For both datasets, we retained six axes, as these axes explained most of the variation attributable to environment (SNP array: *R* ^*2*^ = 0.014 (0.009 MEM corrected), adjusted *R* ^*2*^ = 0.007 (0.002 MEM corrected), *p*‐value <0.001; WGS: *R* ^*2*^ = 0.014 (0.012 MEM corrected), adjusted *R* ^*2*^ = 0.004 (0.003 MEM corrected), *p‐value* <0.001). Temperature associated variables (see heatmap in Supporting information S3) were found to be in line with the primary RDA axis and were the main variation between northern and southern sites (Figure 3). The MEM corrected RDA displayed less separation between northern and southern sites, however, there was still clear distinction between each sample site (Supporting information S11). Three clusters of GO annotations were identified from the Metascape analysis of outlier loci which were associated with phospholipid dephosphorylation, endoderm differentiation, and a combination of notch signaling, axon guidance, and regulation of body fluid levels which are all related to developmental processes.

**FIGURE 4:**
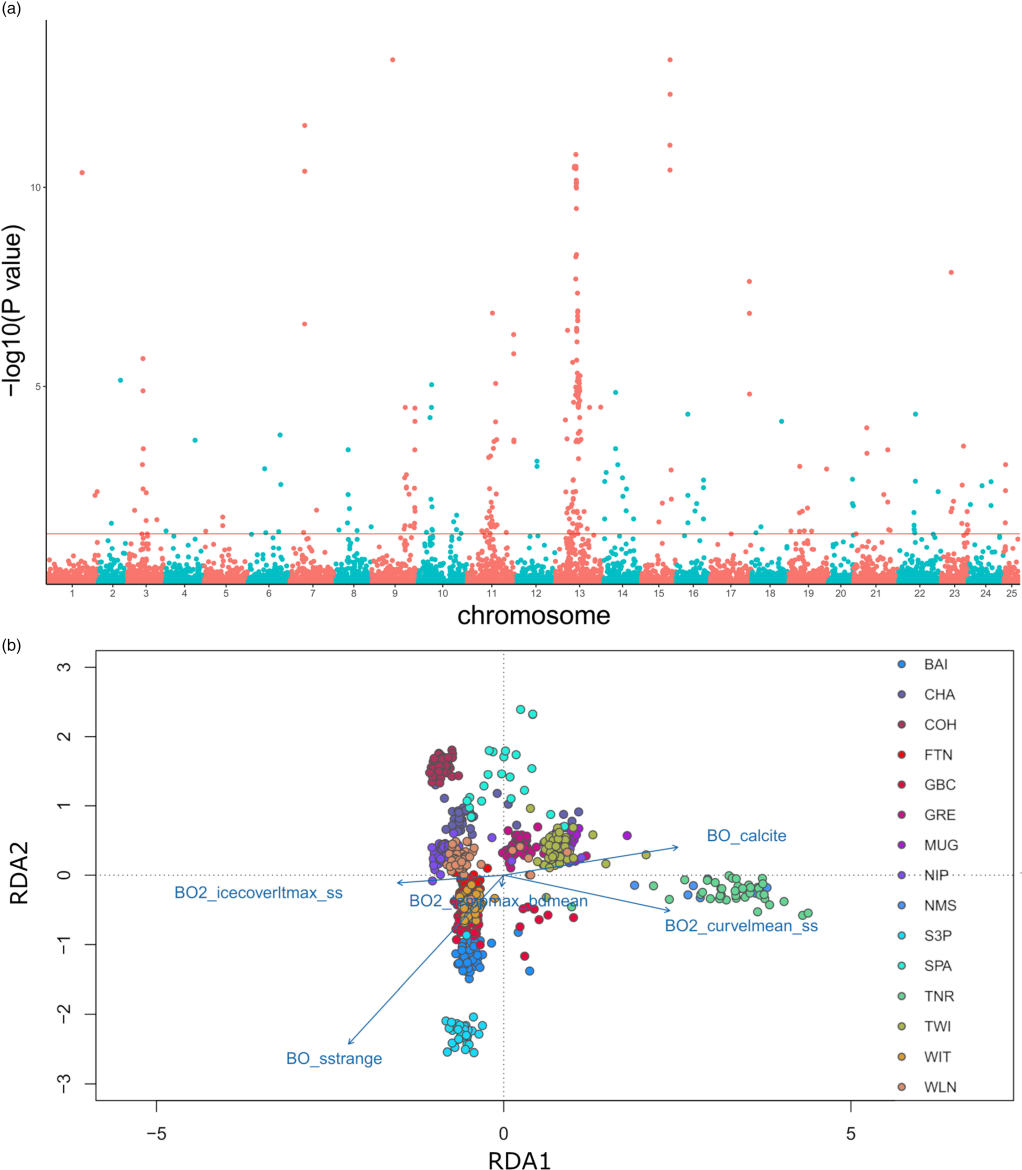
A redundancy analysis (RDA) of all Newfoundland individuals using the whole‐genome re‐sequencing (WGS) data where (a) is a Manhattan plot of the loadings where the red line indicates the threshold in which values above are the top 1% of loci, and (b) is the ordination plot of the first two RDA axis. The *r* ^2^ of the model was 0.012, suggesting the constrained ordination explained approximately 1.2% of the genetic variation. The points represent the individuals colored by their population of origin, while the blue arrows represent the environmental predictors. The relative arrangement of the points and arrows on the plot represents their relationship with the RDA ordination axes.

In order to identify any environmental associations in Newfoundland, we removed the GSL from the north region and re‐ran the RDA. We found five independent variables associated with outlier loci on five significant axes of variation: BO_calcite (281 SNP array loci/1012 WGS loci), BO_sstrange (293/996 loci), BO2_tempmax_bdmean (204/694 loci), BO2_icecoverltmax_ss (131/432 loci), and BO2_curvelmean_ss (267/1218 loci; Figure 4a). Again, we corrected for geography using MEM1 and MEM2 as conditions in the RDA, and identified outlier loci on the same variables: BO_calcite (353 SNP array loci/1151 WGS loci), BO_sstrange (293/1210 loci), BO2_tempmax_bdmean (199/703 loci), BO2_icecoverltmax_ss (95/331 loci), and BO2_curvelmean_ss (287/1521 loci; Supporting information S11). The proportion of explained variance for the SNP array data was *R* ^*2*^ = 0.007 (0.006 MEM corrected), adjusted *R* ^*2*^ = 0.007 (0.001 MEM corrected), *p*‐value <0.001, and for the WGS data was *R* ^*2*^ = 0.008 (0.008 MEM corrected), adjusted *R* ^*2*^ = 0.001 (0.001 MEM corrected), and a *p‐value* <0.001. Both plots produced similar results, where all five variables were significantly correlated with the genetic data and separated each site distinctly along both the primary and secondary axes (Figure 4), implying that environmental factors may be important in the spatial structuring of lumpfish across Newfoundland. We did this analysis with and without chromosome 13 as it contains the sex determining region (Holborn et al., 2022), however, we did not find any difference in result or any loci from the sex determining region in our outlier panel. Using the outlier loci from WGS data, Metascape analysis identified five distinct clusters of annotated genes. Three clusters were related to either cell division, organelle assembly, or endosomal transport. The fourth and fifth clusters of annotated genes were a mix of different biological processes. The fourth cluster was related to both the regulation of bodily fluid levels and the regulation of cell adhesion. The fifth cluster was related to multiple developmental processes and was both the largest cluster of annotated genes and the most interwoven gene network identified.

### 3.5 Comparison of data types

**FIGURE 5:**
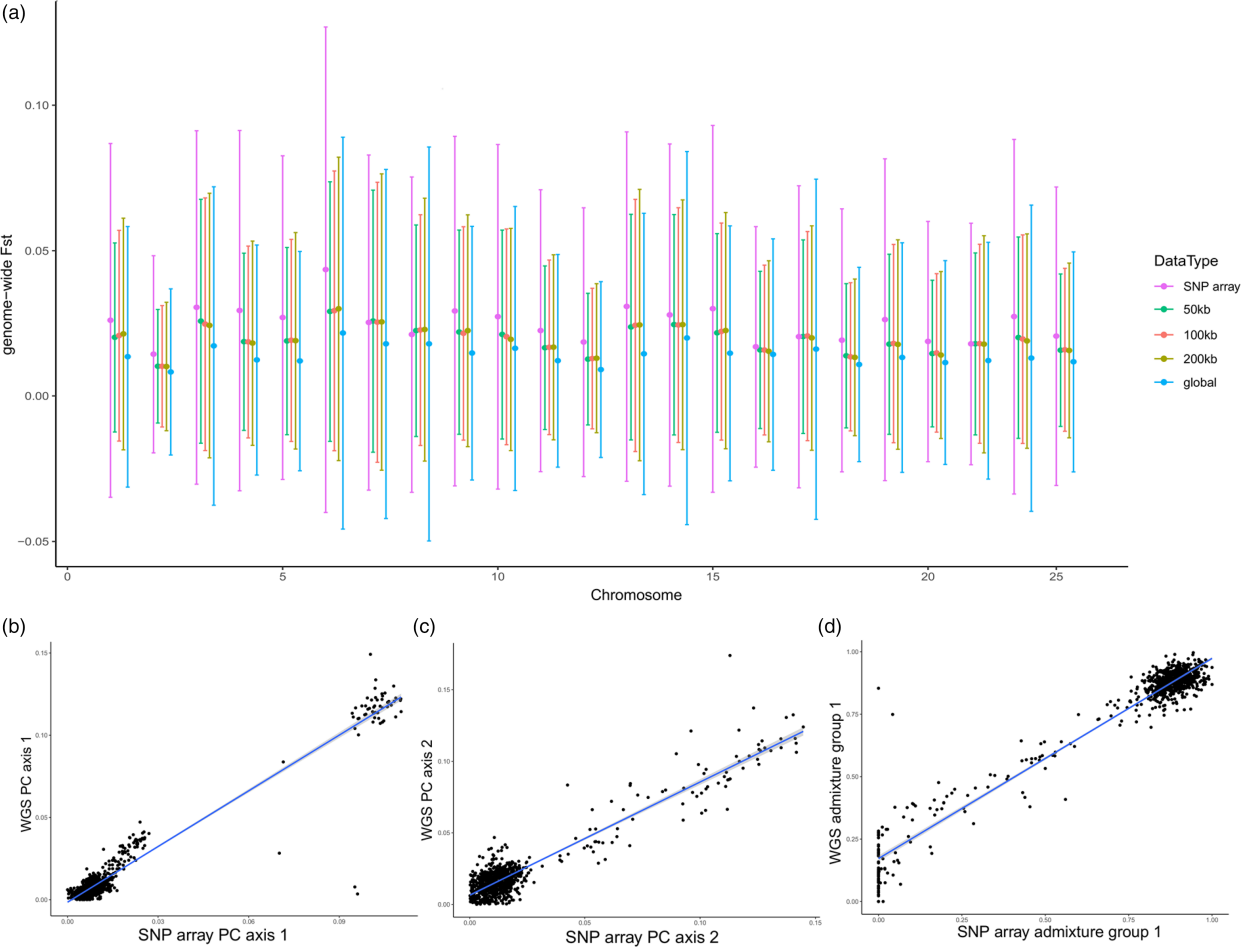
Correlation of SNP array data and whole‐genome re‐sequencing (WGS) data, where (a) is the average by‐loci *F*_ST_ for each chromosome with WGS data split into ascending windows as well as by exomes (i.e., global), (b) is a comparison of the primary principal components axis, (c) is a comparison of the secondary principal components axis, and (d) is a comparison of the first admixture groups (from *K* = 3). WGS data in panel (a) is made up of exomes (244,669 SNPs), while panel (b), (c), and (d) is the entire SNP dataset (5,008,312 SNPs). In panel (a), the dots represent the mean for each chromosome while the bars are the standard deviation around the means. In panels (b), (c), and (d), the blue line of best fit is a linear regression.

Genome‐wide measures of *F*_ST_ comparing the northern to the southern sites between the SNP array and WGS data were significantly correlated (correlation = 0.659, *p*‐value <0.001), as well as when we compared the SNP array to 50 kb WGS windows (correlation = 0.803, *p*‐value <0.001), 100 kb WGS windows (correlation = 0.807, *p*‐value <0.001), and 200 kb WGS windows (correlation = 0.807, *p*‐value <0.001; Figure 5a). When using all loci, the correlation between pairwise *F*_ST_ estimates was 0.965 indicating a high degree of similarity in the population data between datasets (*p*‐value <0.001, 95% CI 0.954–0.974). Similarly, correlations between PC axis 1 (Figure 5b), PC axis 2 (Figure 5c), *K*1 (Figure 5d), *K*2, and *K*3 were all high (Table 2), again consistent with significant similarity among the datasets. However, there were two individuals (see Figure 5b,d) that were not highly correlated; they had the expected assignment based on the SNP array data, however, opposite assignment based on the WGS data. We attribute this difference to an error in the laboratory methods/labeling error as all other individuals are very highly correlated, highlighting the utility of multiple independent datasets for the purpose of error checking.

**TABLE 2:** Correlations between SNP array and whole‐genome re‐sequencing (WGS) data for principal components axes one and two, and all three admixture groups.

Analysis	Correlation	Adjusted *R* ^2^
PC1	0.964	0.930
PC2	0.925	0.856
*K*1	0.969	0.938
*K*2	0.972	0.945
*K*3	0.974	0.949

*Note*: In all cases the adjusted *R* ^2^ was equal to the *R* ^2^, and all *p*‐values were <0.001.

## 4 DISCUSSION

Lumpfish are increasingly in demand as a solution for controlling sea lice in Atlantic salmon farms, yet little is known regarding population structure and the potential impacts of translocation or accidental escapes of farmed lumpfish on wild populations. Using the most extensive genomic survey of spatial variation in this species to date, we identified large‐scale and regional patterns of population structure within this species across the Northwest Atlantic. At large spatial scales, we found a major environmentally associated north–south delineation, with lumpfish from Newfoundland and the GSL forming the north group. Additionally, at finer scales we detected evidence of both discrete groups and isolation by distance around the island of Newfoundland. The consistent evidence of environmental associated differentiation in this species suggests the potential for regional adaptation to play a role in spatial structuring. Overall, the results suggest that lumpfish from Northwest Atlantic appear to be highly structured primarily into North and South groups, mirroring trends observed across other taxa in the region (Benestan et al., 2015; Lehnert et al., 2018; Stanley et al., 2018). The results directly inform conservation plans and utilization strategies for their application in aquaculture as cleaner fish in Atlantic Canada.

### 4.1 Regional differentiation

We found strong evidence to support a north–south delineation of lumpfish in North America. Genome‐wide differentiation supported strong spatial structure between samples from the Gulf of St. Lawrence and Newfoundland and all samples to the south. This result is consistent with previous genomic studies of other species in the region (Benestan et al., 2015; Lehnert et al., 2018; Stanley et al., 2018) again highlighting the potential for a large multi‐species genetic cline in the region. The consistent genetic break found in these studies imply that passive transport by ocean current and associated dispersal mechanisms are unlikely to be driving spatial structure (Stanley et al., 2018), which is likely also the case for lumpfish, despite being semi‐pelagic. Outlier loci between the two regions have functions associated with developmental processes, possibly indicating that lumpfish from either region are evolving to be fundamentally differentiated genome‐wide. Admittedly, our analysis lacks samples from along the Nova Scotia coast which would help resolve the cline in the region of transition. This study extends previous work on lumpfish in the Northwest Atlantic which uncovered a major delineation between Greenland and all sites south of Greenland (Garcia‐Mayoral et al., 2016; Pampoulie et al., 2014), and suggested possible discreteness between the two Gulf of St. Lawrence (Canada) and two Gulf of Maine (USA) sites (Jansson et al., 2022). The accumulating evidence strongly supports regional scale population structure in this species.

Temperature has been found to play a large role in the structuring of marine populations, with the temperature gradient along the Northwest Atlantic providing no exception (e.g., Schmidt et al., 2008). Here, the environmental association analysis suggests significant associations between large‐scale structure and sea temperature and ice cover, providing an ecological mechanism driving differentiation in this species. This is again consistent with previous work on a variety of other species in the area, particularly Stanley et al. (2018) whose multi‐species genomic cline was significantly associated with seasonal temperature minima. These results support hypotheses of broad environmental structuring of marine populations in Atlantic Canada, possibly due to historical vicariance (Bernardi et al., 2003), genetic incompatibilities (Bierne et al., 2011), and/or adaptive divergence due to climate. Climate associated adaptations have been previously found in lumpfish; A similar subdivision of lumpfish has been suggested in the east Atlantic where lumpfish from the English Channel have a distinct genetic break from more northernly populations. These lumpfish spawn earlier due to warmer temperatures and increased food availability (Hedeholm et al., 2017). Similar ecological mechanisms could be at play here along the Northwest Atlantic coast and warrant further examination.

### 4.2 Cryptic differentiation

Beyond the North–South subdivision, our results suggest significant non‐spatial limits to gene flow within the Northern region, reflected in the detection of two population clusters within multiple sites. Interestingly, the presence of a discrete population represented primarily by several non‐proximate juvenile samples with some adults from the around the island of Newfoundland was unexpected. Moreover, genome‐wide evaluation of differences between these groups suggests that the differences are widespread across the genome and significant. The presence of this discrete group could not be explained by relatedness or sex‐biased sampling and was present both in the SNP array and whole‐genome re‐sequencing datasets (Table 1 and Table 2). As such, it supports a hypothesis of a discrete and cryptic coastal life history form present in embayments of Newfoundland's north and south coasts. Marine species, such as Atlantic cod (*Gadus morhua*), haddock (*Melanogrammus aeglefinus*), and northern shrimp (*Pandalus borealis*) have also reported genetically different inshore and offshore groups (Berg et al., 2021; Hansen et al., 2021; Johansen et al., 2018; Pampoulie et al., 2011; Ruzzante et al., 2011), and in the case of cod, also falling along the Newfoundland coast. The environmental association analysis of the northern region further suggests that oceanographic dispersal may be involved in the isolation of this coastal group, therefore, implying they may be localized to inshore retentive conditions. This is consistent with suggestions of phenotypic differentiation in lumpfish elsewhere including reports of presence in low‐salinity habitats such as Hudson Bay and the Baltic Sea (Davenport, 1985; Powell et al., 2018) and ecological differences in the warmer conditions of the English Channel (Hedeholm et al., 2017). Nonetheless, further genomic and behavioral studies are warranted to evaluate this hypothesis for this group in Newfoundland and separate management strategies could be utilized when dealing with the offshore as opposed to onshore lumpfish.

### 4.3 Fine‐scale environmental differentiation

In the primary northern group, we found significant genetic structuring based on environmental variables and a positive isolation by distance. We found discretely structured sampling sites based on a variety of environmental variables, suggesting that localized adaptation could be occurring. Therefore, site fidelity of adults and their life history/local oceanographic conditions seems to allow the isolation of lumpfish across large and small spatial scales. A recent study found that sampling that took place at breeding sites or within juvenile groups, formed clear‐cut groups, while sampling of actively migrating individuals were more admixed (Jansson et al., 2022), implying that discrete population structure could potentially be linked to homing behavior. Lumpfish have several life history traits aside from homing that could also contribute to local adaptation, including eggs that are generally attached to an inshore substrate, such as rocks (Goulet & Green, 1988), a juvenile stage which adheres to inshore seaweed by way of their pelvic adhesive disk for up to a year (Sheehan et al., 2012; Vandendriessche et al., 2007), and an adult stage which often adheres to solid objects such as lobster pots and rocks to keep from drifting (COSEWIC, 2017). The tendency of lumpfish to adhere to objects throughout their life may play a large role in environmentally shaping populations. Based on the current datasets, we were unable to observe any fine‐scale structuring in the southern region, and it is possible that the semi‐pelagic nature of lumpfish could play a larger role in homogenizing lumpfish throughout the warmer southern region.

### 4.4 Management of lumpfish across their North American range

Lumpfish are of growing commercial interest for their ability to effectively reduce sea lice in salmon farms (Deady et al., 1995; Groner et al., 2013; Treasurer, 2012). A genomic understanding of structuring in this species as presented here could inform both exploitation strategies, as well as the mitigation of genetic impacts of escaped translocated individuals used in salmon aquaculture (Whittaker et al., 2018). Despite the large trans‐Atlantic range and high dispersal migration ability of lumpfish, they may have low resilience to fishing pressure due to their trophic level and long population doubling time (between 4.5 and 14 years; Powell et al., 2018). Currently, lumpfish across Eastern Canada are grouped together as one conservation unit (e.g., DU or ESU) (Simpson et al., 2016). However, our analyses suggests that two to three distinct and adaptively discrete groups are present. As such, the translocation of lumpfish across these major regional zones could have negative effects on the local populations resulting from introgression with escapees (Blakeslee et al., 2020; Glover et al., 2012). In recent years, escapees from salmon aquaculture have been suggested to negatively impact wild populations by transmitting disease (Bjørn & Finstad, 2002; Heuch & Mo, 2001; Krkošek et al., 2007; Skilbrei & Wennevik, 2006), outcompeting wild fish for resources (Jensen et al., 2010), and reducing genetic integrity and overall fitness of wild populations (Baskett et al., 2013; Fleming et al., 2000; Solberg et al., 2016). Despite the wide‐ranging distribution of lumpfish, the presence of discrete populations in Newfoundland and elsewhere (Davenport, 1985; Hedeholm et al., 2017; Powell et al., 2018) strongly suggests regional‐based management and conservation to preserve adaptive diversity in this species across the North Atlantic.

### 4.5 Whole‐genome data approach to marine studies

This study provided a unique opportunity to directly compare the results of SNP array and whole‐genome sequence data for the inference of population structure in a marine fish. The purpose of this comparison was to evaluate if both datasets were consistent and able to resolve similar patterns of genomic and population structure. The SNP array utilized was ascertained using European lumpfish (Knutsen et al., 2018) and as such is likely subject to ascertainment bias and may underestimate diversity in the Northwest Atlantic (e.g., Bradbury et al., 2011). Similarly, WGS data can suffer from both individual level and site level quality and coverage variation which can also bias inference (Gopalakrishnan et al., 2022; Lou et al., 2021; Nielsen et al., 2011). Despite these potential limitations, our results suggest highly significant similarities in the population analysis results between WGS data and SNP array data. The implications of the observed similarity are twofold. First, these results suggest that the influence of any ascertainment bias in the utilization of the SNP array in the Northwest Atlantic are minimal and has not impacted the detection of broad scale patterns in population structure. Secondly, we observe that WGS data accurately captures patterns in population structure and does so with high levels of genomic coverage. Accordingly, this work adds to a growing body of research suggesting that moderate to low‐depth whole‐genome sequence data are highly applicable to the resolution of population structure and adaptation in the wild (Fuller et al., 2020; Kess, Dempson, et al., 2021; Kess, Einfeldt, et al., 2021; Wilder et al., 2020). Admittedly, WGS data may suffer possible calling errors due to the uneven coverage and imputation of missing loci (see Fuentes‐Pardo & Ruzzante, 2017). Based on the strong significant correlation between the SNP array and WGS data here, error associated with either method do not appear to have significantly impacted population structure estimates, and the comparison highlights the utility of validating population genomic inferences with multiple genotyping methods.

## 5 CONCLUSIONS

Our results suggests that previously unrecognized regional patterns of population structure of lumpfish across the Northwest Atlantic are significantly driven by environmental associations. Regional patterns include the presence of a major north–south delineation as noted for other species in the region, as well as further structuring within the northern area. The comparison of the SNP array and whole‐genome re‐sequencing data further supports the use of low‐depth whole‐genome data as a viable and effective approach for population genomics of marine species. The results directly inform the further conservation of this at‐risk species as well as its continued development as a cleaner fish in salmonid aquaculture. Despite the wide‐ranging distribution of lumpfish, the presence of discrete populations in Newfoundland and elsewhere (Davenport, 1985; Hedeholm et al., 2017; Powell et al., 2018) suggests that regional management and conservation of lumpfish is warranted to preserve adaptive diversity in this species across the North Atlantic.

## CONFLICT OF INTEREST STATEMENT

We declare no conflicts of interest.

## Supporting information


Data S1:

## Data Availability

Datafiles used in this study are available on Dryad at: DOI https://doi.org/10.5061/dryad.q83bk3jpq.
