# Supplementary material for: Fine‐scale environmentally associated spatial structure of lumpfish (Cyclopterus lumpus) across the Northwest Atlantic
Source: Evol Appl. 2023 Sep 5;16(9):1619–36. doi: 10.1111/eva.13590 (PMC10519416; doi:10.1111/eva.13590)
Supplement: Supplementary file 1 — Data S1: [file EVA-16-1619-s001.zip › Langille_etal_2023_SupplementaryInformation1to11.docx]

*Evolutionary Applications*

**Fine-scale environmentally associated spatial structure of Lumpfish (*Cyclopterus lumpus*) across the Northwest Atlantic**

Barbara L. Langille, Tony Kess, Matthew Brachmann, Cameron M. Nugent, Amber Messmer, Steven J. Duffy, Melissa K. Holborn, Mallory Van Wyngaarden, Tim Martin Knutsen, Matthew Kent, Danny Boyce, Robert S. Gregory, Johanne Gauthier, Elizabeth A. Fairchild, Michael Pietrak, Stephen Eddy, Carlos Garcia de Leaniz, Sofia Consuegra, Ben Whittaker, Paul Bentzen, Ian R. Bradbury

Supplementary Information (S1 to S11)

Supplementary 1: Summary table of all sample sites ordered alphabetically in each sampling region. Each region is in order by decreasing latitude. Population codes in bold, denote samples that have both SNP array data and whole genome re-sequencing data. Population codes with an asterix (*), denote sample sites that consist of juveniles.

| Region | Location | Population | Latitude | Longitude | N |
| --- | --- | --- | --- | --- | --- |
| Newfoundland | Baine Harbour | **BAI** | 47.17737 | -54.8447 | 55 |
|  | Baine Harbour | **BHbr** | 47.35687 | -54.8897 | 48 |
|  | Witless Bay | CA | 47.28127 | -52.788 | 14 |
|  | Champneys | **CHA** | 48.36933 | -53.2911 | 46 |
|  | Cooks Harbour | **COH** | 51.59933 | -55.5864 | 50 |
|  | Fortune | **FTN** | 47.08832 | -55.823 | 98 |
|  | Gooseberry Cove | **GBC** | 48.0302 | -53.6243 | 98 |
|  | Greenspond | **GRE** | 49.06475 | -53.5735 | 58 |
|  | Musgrave Harbour | **MUG** | 49.46722 | -53.9175 | 50 |
|  | Nippers Harbour | **NIP** | 49.78744 | -55.8486 | 50 |
|  | Newman Sound | **NMS*** | 48.55123 | -53.962 | 10 |
|  | Multi-species Survey 3Ps | **S3P** | 46.5275 | -55.0983 | 35 |
|  | Placentia Bay | **SPA*** | 47.19486 | -55.0746 | 22 |
|  | Terra Nova | **TNR*** | 48.6541 | -53.565 | 50 |
|  | Twillingate | **TWI** | 49.71236 | -54.7705 | 50 |
|  | Witless Bay | **WIT** | 47.28127 | -52.788 | 118 |
|  | Wild Cove | **WLN** | 49.99576 | -56.3535 | 59 |
| The Gulf of St. Lawrence | 1 | **TE** | 49.5385 | -66.5285 | 2 |
|  | 2 | **TE** | 49.68483 | -64.28217 | 1 |
|  | 3 | **TE** | 49.94533 | -66.7712 | 2 |
|  | 4 | **TE** | 49.8715 | -66.2905 | 1 |
|  | 5 | **TE** | 49.9445 | -65.7805 | 1 |
|  | 6 | **TE** | 49.72033 | -64.7538 | 1 |
|  | 7 | **TE** | 50.22 | -64.331 | 1 |
|  | 8 | **TE** | 50.07433 | -65.744 | 5 |
|  | 9 | **TE** | 49.03633 | -63.2387 | 3 |
|  | 10 | **TE** | 48.927 | -62.2175 | 1 |
|  | 11 | **TE** | 50.53183 | -57.5828 | 1 |
|  | 12 | **TE** | 50.63767 | -57.8573 | 1 |
|  | 13 | **TE** | 49.93533 | -65.597 | 2 |
|  | 14 | **TE** | 50.07433 | -65.744 | 2 |
|  | 15 | **TE** | 50.1685 | -58.9112 | 1 |
|  | 16 | **TE** | 50.01517 | -62.2098 | 1 |
|  | 17 | **TE** | 49.65917 | -60.3698 | 1 |
|  | 18 | **TE** | 50.072 | -61.0867 | 1 |
|  | 19 | **TE** | 49.58867 | -66.23217 | 1 |
|  | 20 | **TE** | 49.67717 | -66.8175 | 2 |
|  | 21 | **TE** | 49.93533 | -65.597 | 3 |
|  | 22 | **TE** | 49.8345 | -59.6395 | 1 |
|  | 23 | **TE** | 50.07533 | -63.4533 | 1 |
|  | 24 | **TE** | 50.09417 | -62.5688 | 1 |
|  | 25 | **TE** | 50.01517 | -62.2098 | 1 |
|  | 26 | **TE** | 50.02883 | -63.718 | 2 |
|  | 27 | **TE** | 48.11017 | -60.7257 | 2 |
|  | 28 | **TE** | 48.29033 | -60.6043 | 1 |
|  | 29 | **TE** | 49.1885 | -61.223 | 3 |
|  | 30 | **TE** | unknown | unknown | 1 |
|  | 31 | **TE** | 49.16883 | -67.8075 | 1 |
|  | 32 | **TE** | 47.87467 | -60.4605 | 1 |
|  | 33 | **TE** | 47.74167 | -60.59 | 1 |
|  | 34 | **TE** | 49.47833 | -59.3395 | 1 |
|  | 35 | **TE** | 51.3945 | -56.76767 | 1 |
|  | 36 | **TE** | 51.49333 | -56.43917 | 1 |
|  | 37 | **TE** | 50.57183 | -58.08617 | 1 |
|  | 38 | **TE** | 50.23583 | -58.614 | 1 |
|  | 39 | **TE** | 50.08433 | -58.9475 | 1 |
|  | 40 | **TE** | 49.65917 | -60.3698 | 1 |
|  | 41 | **TE** | 49.506 | -61.17933 | 1 |
|  | 42 | **TE** | 50.05067 | -62.69417 | 5 |
|  | 43 | **TE** | 49.9935 | -64.3235 | 4 |
|  | 44 | **TE** | 49.95067 | -64.99033 | 6 |
|  | 45 | **TE** | 50.18083 | -65.396 | 9 |
|  | 46 | **TE** | 50.1415 | -65.82283 | 3 |
|  | 47 | **TE** | 49.97667 | -65.58717 | 6 |
|  | 48 | **TE** | 49.80483 | -65.2425 | 3 |
|  | 49 | **TE** | 49.63783 | -60.77167 | 1 |
|  | 50 | **TE** | 49.71883 | -66.989 | 3 |
|  | 51 | **TE** | 49.8905 | -66.4115 | 1 |
|  | 52 | **TE** | 49.71367 | -66.4145 | 1 |
|  | 53 | **TE** | 49.557 | -60.9962 | 1 |
|  | 54 | **TE** | 48.84483 | -67.77083 | 1 |
|  | 55 | **TE** | 48.82367 | -67.96817 | 1 |
|  | 56 | **TE** | 49.69883 | -66.83017 | 2 |
|  | 57 | **TE** | 49.54367 | -63.95367 | 2 |
|  | 58 | **TE** | 49.55633 | -66.72383 | 2 |
| New Brunswick | Pond Point Grand Manan | **PPG** | 44.6254 | -66.8006 | 13 |
| United States | Bay East of Beals Island | **BIM** | 44.51262 | -67.6018 | 16 |
|  | Frenchmans Bay | **FBM** | 44.42487 | -68.2372 | 29 |
|  | Scantums Basin | **MSB** | 42.83333 | -70.5 | 17 |
|  | Cobscook Bay | US1 | 44.90583 | -67.0733 | 15 |
|  | Frenchmans Bay | US2 | 44.42487 | -68.2372 | 14 |

Supplementary 2: Additional Methods and Results for the Gulf of St. Lawrence.

*Additional Methods*

We had a total of 58 different sampling sites across the entire Gulf of St. Lawrence (GSL) region (Fig.1; S1), however, we only had between one and nine individuals per site. Therefore, in order to use the GSL samples in our dataset, we needed to determine a different grouping of individuals as many analyses can be biased by small samples sizes (Lin 2018). We calculated a neighbor joining tree in R of the GSL individuals, using the package *StAMPP* (*stamppNeisD*), which generated a Nei’s D distance matrix, while the *ape* package (*nj*) was used to build the tree using default settings. We used FigTree v.1.4.3 (Rambaut 2010) to visualize the phylogeny. We computed pairwise *F*_ST_ in ARLEQUIN (Excoffier and Lischer 2010) between sites from the GSL in order to determine functional groupings for downstream analyses. Finally, we also visualized individuals using a multivariate exploratory approach: Principal Components Analysis (PCA), which estimates genomic variation between individuals on uncorrelated axes, and non-negative matrix factorization to directly infer admixture proportions, using the package *pcadapt* in R. The number of components was determined by a scree-plot, which used a value of *K* to identify the correct number of principal components. We calculated PC scores for a K=10 and min.maf of 0.01.

*Additional Results*

In order to use samples collected from the GSL in all analyses, we combined sample sites into larger groups (i.e. more than 10 individuals per group) based on a combination of geographically proximal groups as well as on the following analyses. No majorly divergent groups were identified by a neighbor-joining tree, however, seven smaller clusters did emerge (S2a). Sites to the far northeast of the GSL were found at the bottom of the phylogeny as indicated by blue dots (sites 36 and 37), while sites in the western part of the range were found at the top of the phylogeny as indicated by the purple dots (sites 55 and 56). Individuals found in the southernmost part of the GSL (sites 33 and 34), as indicated by green dots, were positioned closer to the northeast sites in the phylogeny. Pairwise *F*_ST_ between sample sites revealed that most individuals were quite similar as they had p-values of > 0.999 (S2b). However, there were a group of sites, 31, 43 to 49, and 58, that were slightly differentiated from most other sites, although not significantly so as the p-values generally did not fall under 0.05. These slightly divergent sites were also different from each other and correlated loosely with the middle section of the phylogeny. There were only six significantly differentiated sites in the entire GSL range (43-10, 43-15, 43-28, 43-31, 46-15, and 46-49). Sites 43 and 46 were found in approximately the middle of all the GSL sites geographically and in the phylogeny (represented by black and red dots respectively), therefore we were able to use the few sites they were most differentiated from as break points. We plotted PCA first with all GSL sites labeled individually, and again with GSL sites labeled approximately based on clustering like-sites together. In the first PCA, a mixed cline of sites moving along the secondary axis was evident (S2c). The majority of variation was on the first axis, however, only site 43 clustered apart from all others. which implied most individuals were quite similar in structure (1.77% explained variance on axis 1 and 1.24% of axis 2), as was also evident by the phylogeny and the pairwise *F*_ST_ estimates. Based on these analyses and the physical distance between sites, we were able to delineate the GSL into three regions: western GSL (TE1), central GSL (TE2), and eastern GSL (TE3) (Fig 1; S2d for map of the GSL sites placed into the same groups). These new groups were used in all analyses moving forward.


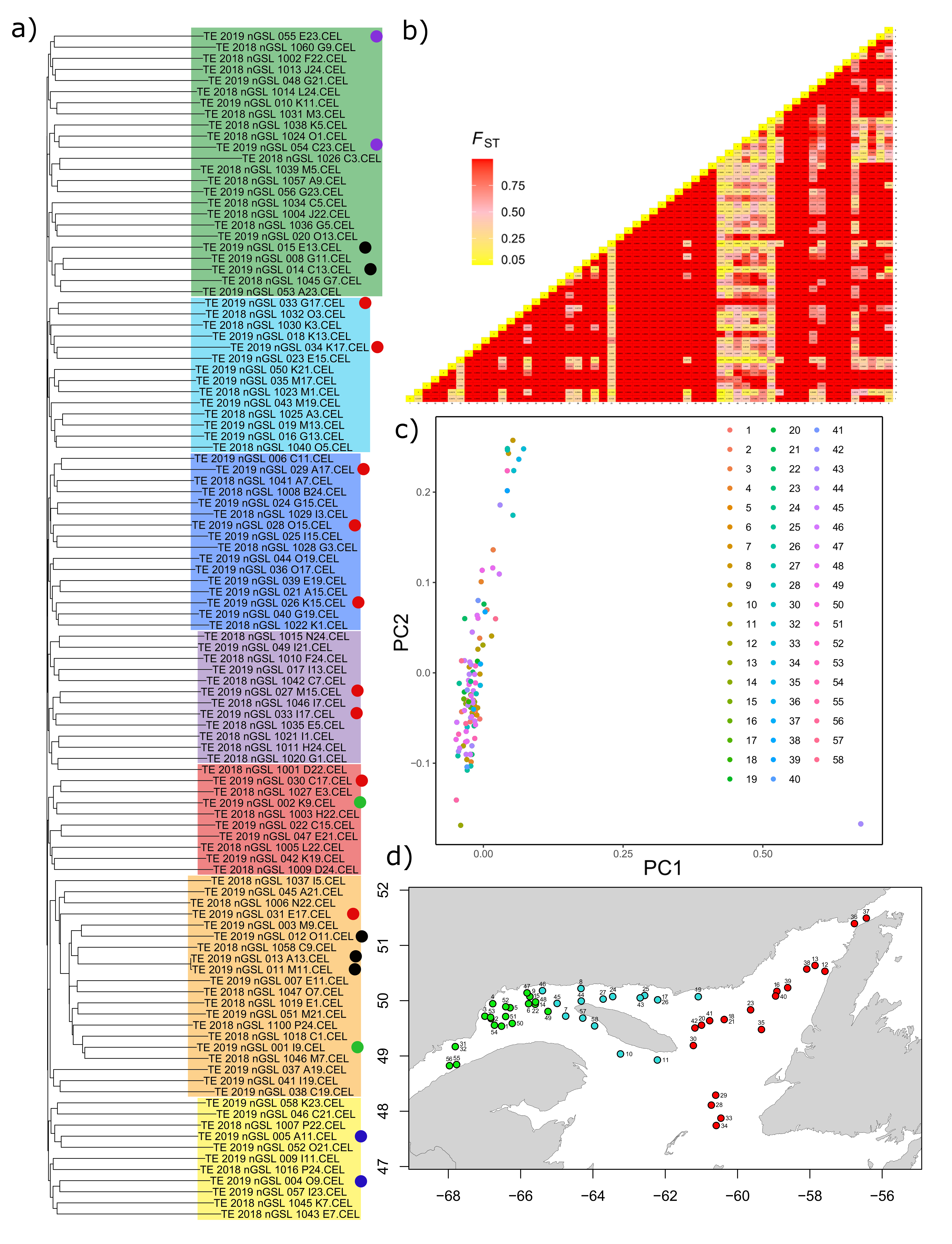


S2 Figure: (a) is a Neighbor Joining tree of all Gulf of St. Lawrence (GSL) samples, where colored blocks represent different proposed groups based on Nei’s D distances. Colored blocks separate groups based generally on those with closest assignment in the phylogeny, however, do not comment on the nature of the individuals found inside each block. The colored circles represent: blue - sites 36 and 37 which are the furthest northeast sites, purple - sites 55 and 56 which are the furthest western sites, green - sites 33 and 34 which are the furthest southern sites, black - site 43 and red - site 46, which were both identified by pairwise *F*_ST_ (S5) to be the only two sites that were significantly differentiated from other sites. (b) is a pairwise *F*_ST_ plot of *p*values for GSL populations (labelled 1through 58 - see map for exact placement though the GSL). The red cells indicate sample sites that are not significantly different from each other, while the yellow cells indicate the sites are very different. (c) is a PCA plot of the GSL region sampling sites using the site numbers (see S1 for exact placement). Finally, (d) is an updated map of GSL region with proposed populations based on pairwise *F*_ST_, neighbor joining tree, and PCA plot.


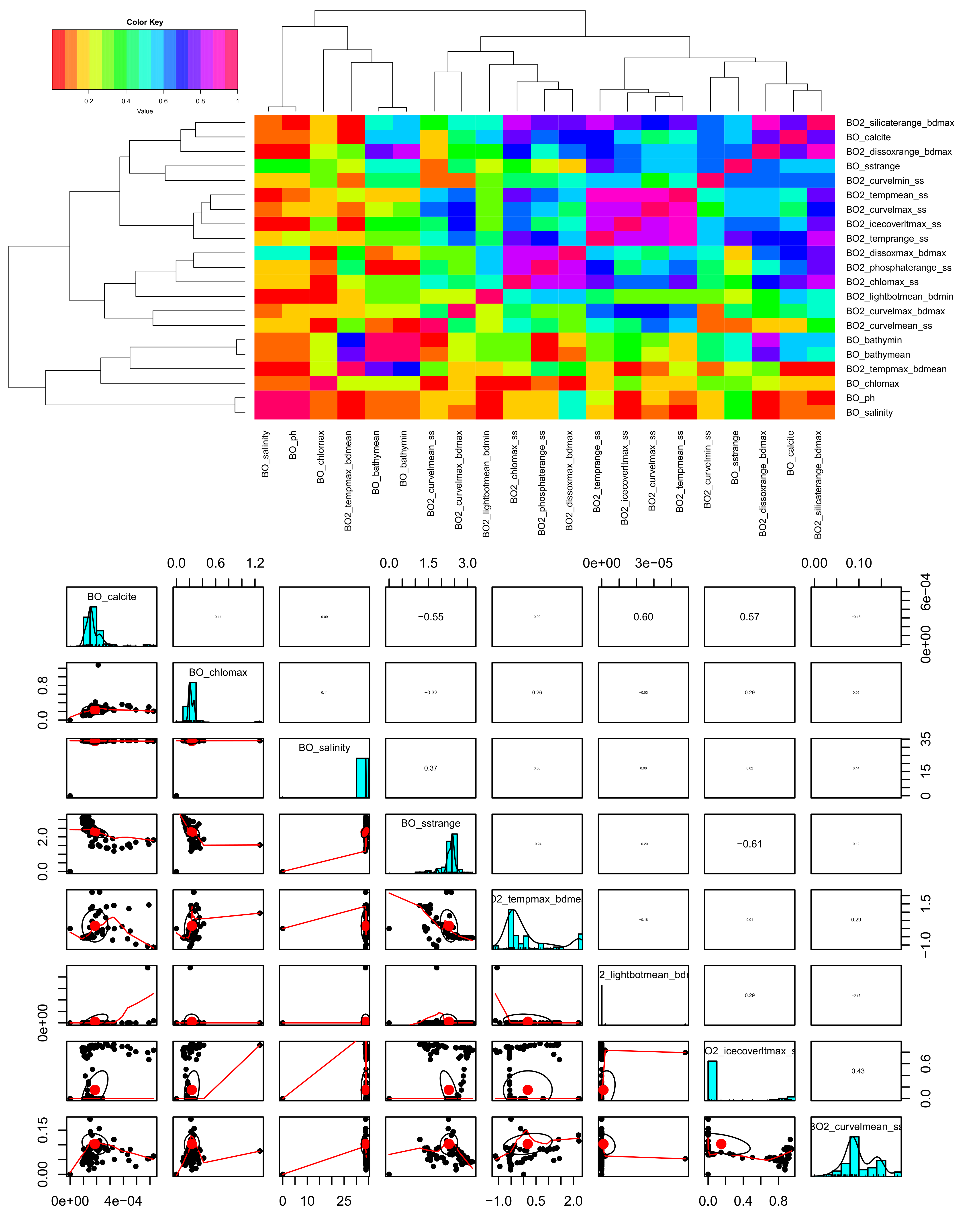


Supplementary 3: Heatmap of correlated variables using in the Redundancy Analysis (RDA), where blacks of dark blue, purple, and magenta indicate high collinearity. The lower panel is the pairs.panels plot where plots above the diagonal are the Pearson correlations, bivariate scatter plots are below the diagonal, and histograms on the diagonal.

Supplementary 4: See attached file of all outlier loci from PCAs and RDAs.


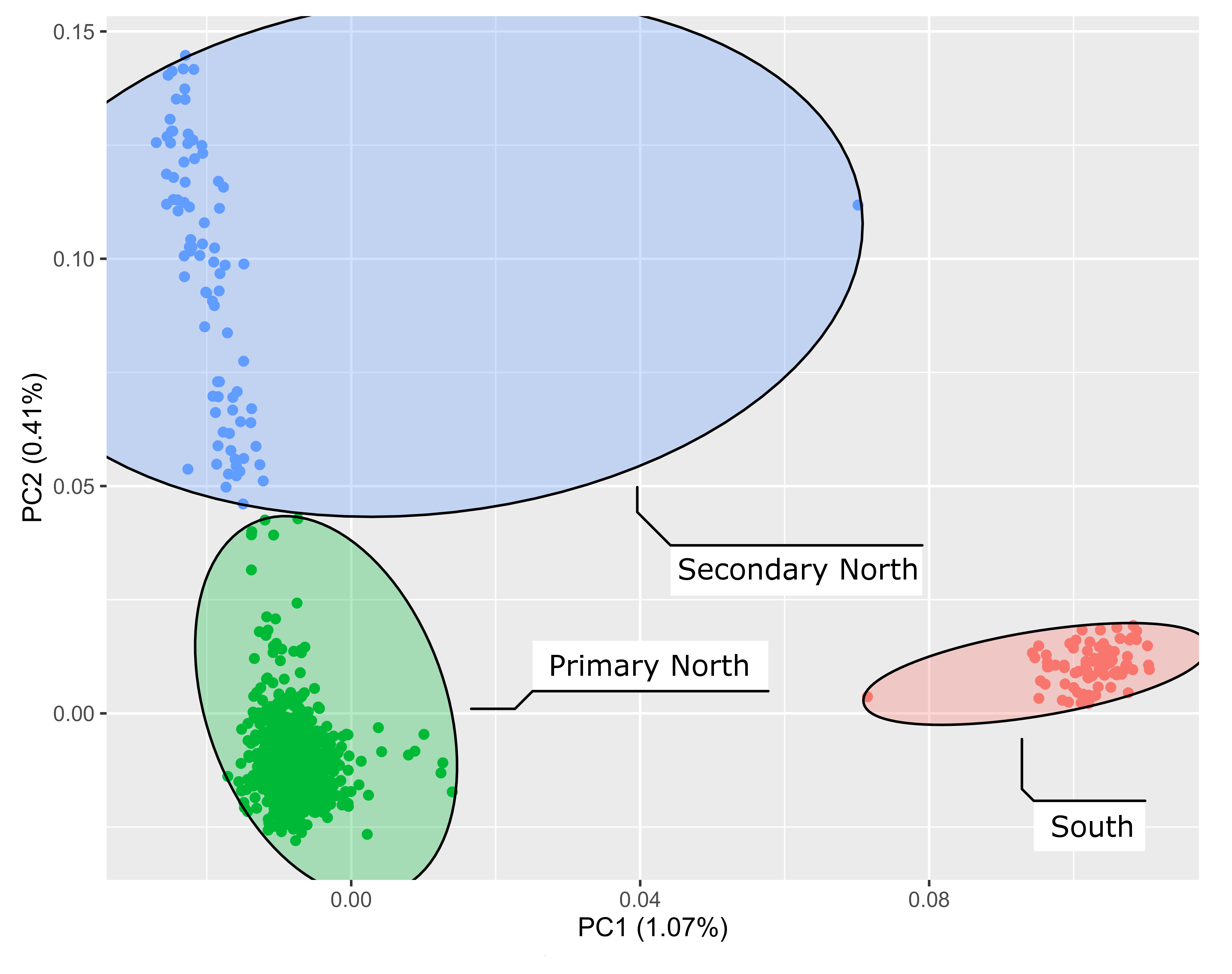


Supplementary 5: *K*-means clustering of all North American sites where three groups were supported with separation into: a distinct south group (Grand Manan and Gulf of Maine), primary north group (Newfoundland adults and Gulf of St. Lawrence), and a secondary north group (Newfoundland juveniles and some Newfoundland adults from a variety of sample sites).


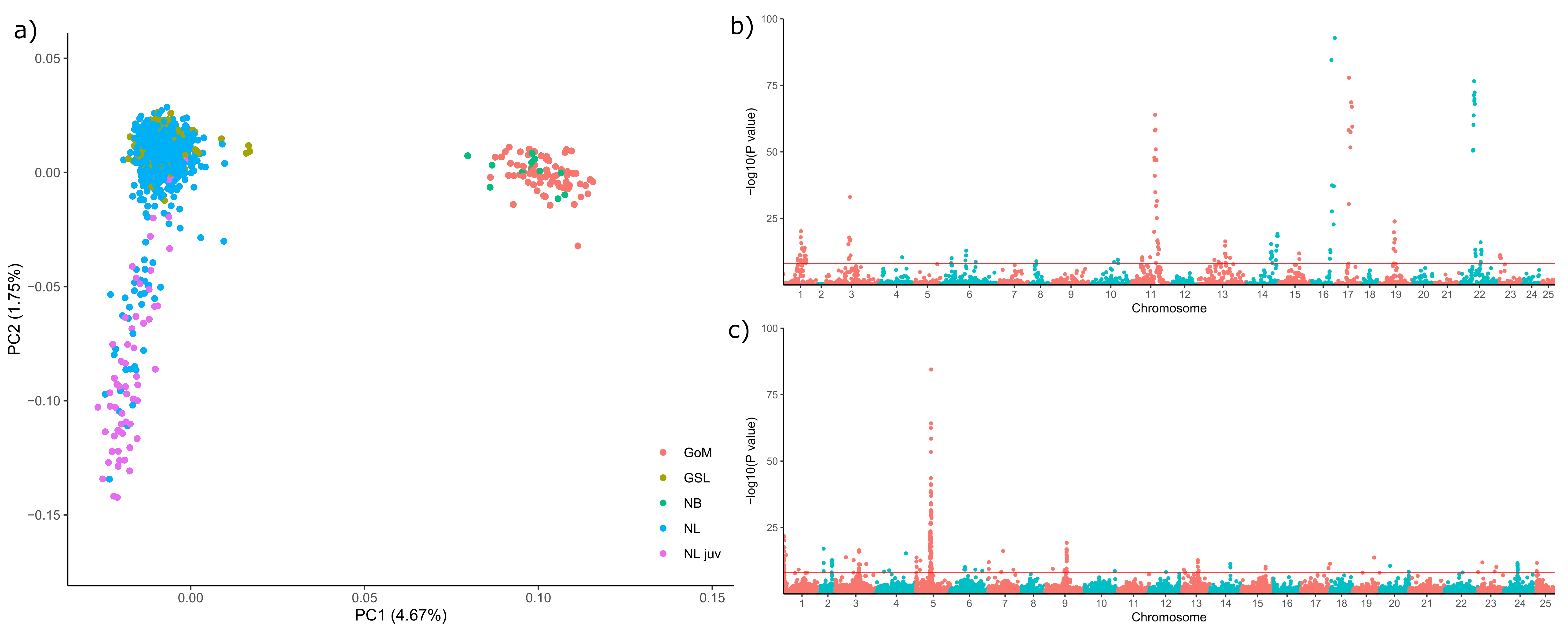


Supplementary 6: PCadapt plot of all sample sites across the Northwest Atlantic Ocean where a) is the result with only qvalue outlier loci, b) is the loadings of the qvalue outlier loci, and c) is the loadings of the neutral loci.


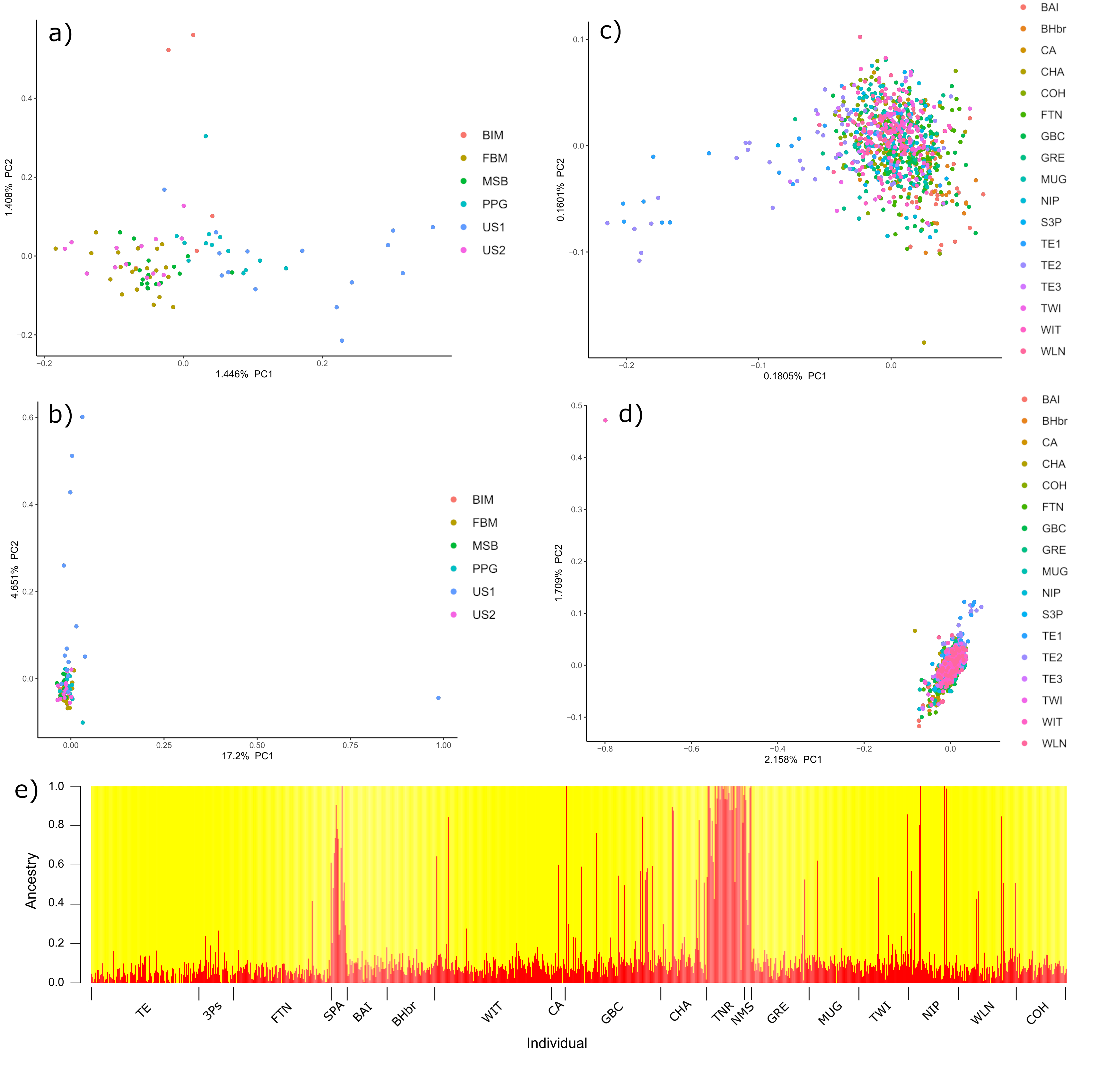


Supplementary 7: Principal components analysis (PCA) of neutral (a) and outlier (b) loci from the south range, and neutral (c) and outlier (d) loci from the north range. (E) is an admixture plot of the north range, highlighting the primary and secondary clusters.


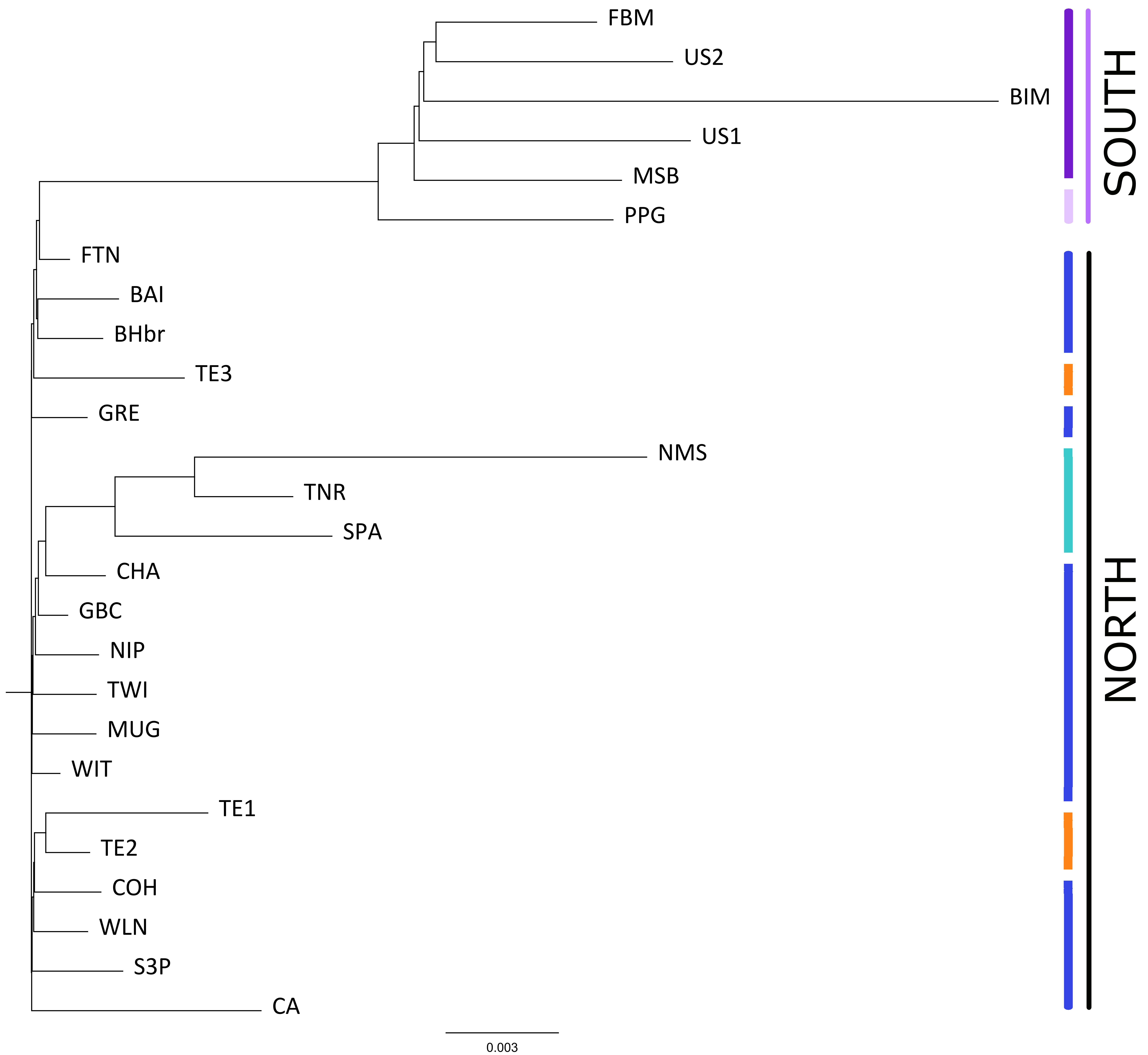


Supplementary 8: Neighbor-joining tree of all sites which split into north (Newfoundland and GSL) and south (Grand Manan and the Gulf of Maine) groups. Within the south group, the dark purple line represents sites in the Gulf of Maine, and the light purple line represents the one site in Grand Manan. Within the north group, the dark blue lines represents adult Newfoundland sites, the light blue line represents juvenile sites in Newfoundland, and the orange line represents the sites in the GSL.


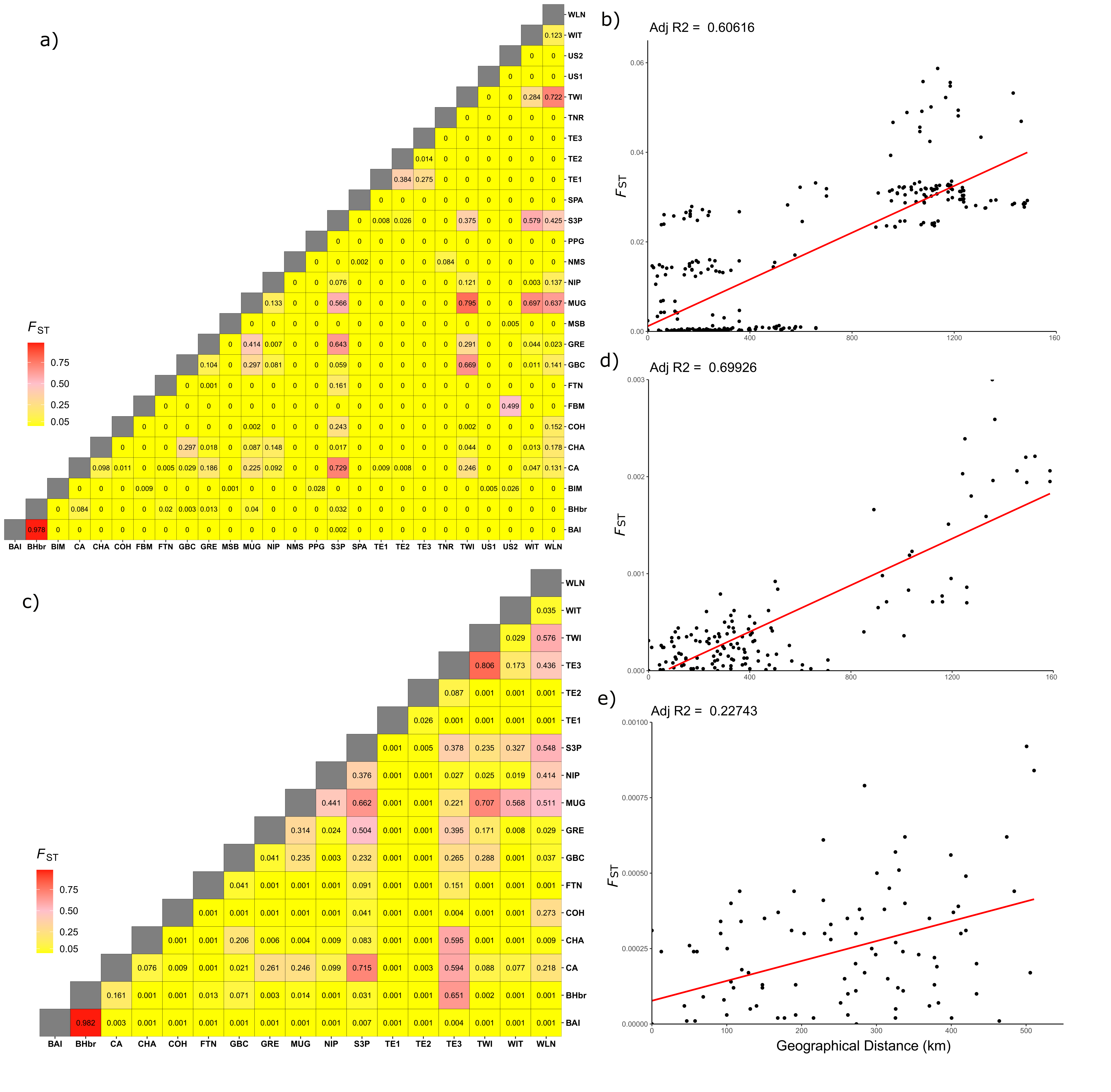


Supplementary 9: (a) is a plot of pairwise *F*_ST_ comparisons of all sites where all *p* values are represented in this table, (b) is the corresponding isolation by distance (IBD) plot where pairwise *F*_ST_ is calculated between all sample sites and plotted against distance, (c) pairwise *F*_ST_ comparison of all sites in the primary north group (i.e. south and secondary groups removed) where all *p* values are represented in this table, (d) is an IBD plot all sample sites in the primary north group, and (e) is an IBD plot of Newfoundland adults. In all IBD plots the regression line in red indicates the slope of all points, linking ~ 60.6%, 69.9%, and 22.7% of the genetic data to geographic distance, respectively.

Supplementary 10: See attached text file for confidence intervals for pairwise *F*_ST_.


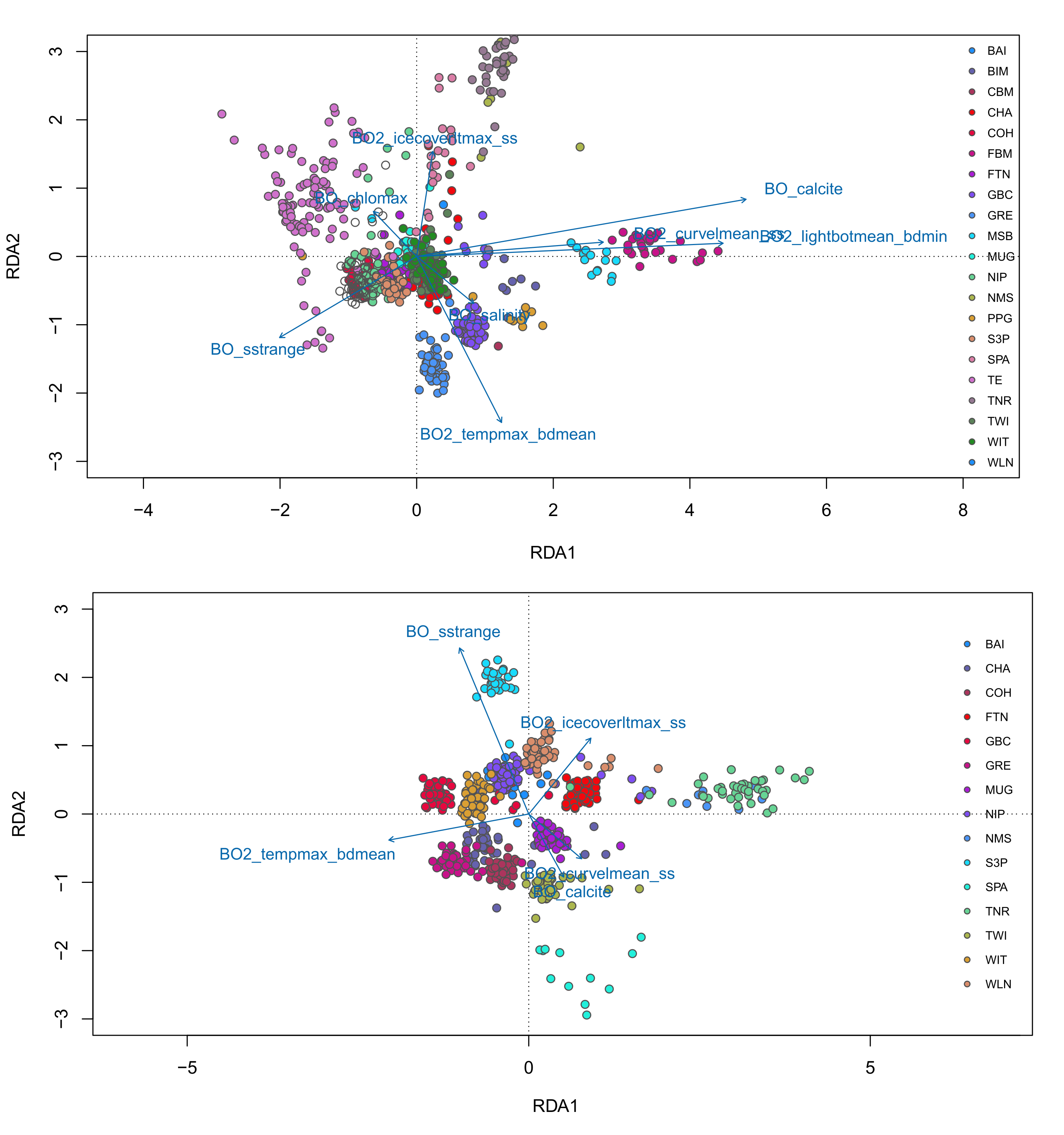


Supplementary 11: MEM corrected RDAs from the WGS data where the top plot includes all individuals across North America and the bottom plot is only individuals across Newfoundland.
